# Long non-coding RNA, HOTAIRM1, promotes glioma malignancy by forming a ceRNA network

**DOI:** 10.18632/aging.102205

**Published:** 2019-09-02

**Authors:** Qingyu Liang, Xue Li, Gefei Guan, Xiaoyan Xu, Chen Chen, Peng Cheng, Wen Cheng, Anhua Wu

**Affiliations:** 1 Department of Neurosurgery, The First Hospital of China Medical University, Shenyang, Liaoning Province, China; 2 Department of Pathophysiology, College of Basic Medicine Science, China Medical University, Shenyang, Liaoning Province, China; 3 The Research Center for Medical Genomics, Key Laboratory of Cell Biology, Ministry of Public Health, Key Laboratory of Medical Cell Biology, Ministry of Education, College of Life Sciences, China Medical University, Shenyang, Liaoning Province, China

**Keywords:** glioma, HOTAIRM1, prognosis, ceRNA network, immune microenvironment

## Abstract

Long non-coding RNAs play critical roles in tumorigenesis and the immune process. In this study, RNA sequencing data for 946 glioma samples from The Cancer Genome Atlas and the Chinese Glioma Genome Atlas databases were analyzed to evaluate the prognostic value and function of homeobox A transcript antisense RNA myeloid-specific (HOTAIRM)1. HOTAIRM1 expression was associated with clinical and molecular features of glioma: patients with high HOTAIRM1 expression were more likely to be classified as malignant cases, and elevated HOTAIRM1 level was associated with shorter survival time in subgroups stratified by clinical and molecular features. A multivariate Cox regression analysis showed that HOTAIRM1 was an independent prognostic factor for patient outcome. In vitro experiments revealed that HOTAIRM1 knockdown suppressed the malignant behavior of glioma and increased tumor sensitivity to temozolomide. The results of an in silico analysis indicated that HOTAIRM1 promotes the malignancy of glioma by acting as a sponge for microRNA (miR)-129-5p and miR-495-3p. HOTAIRM1 overexpression was also associated with immune activation characterized by enhanced T cell-mediated immune and inflammatory responses. These results suggest that HOTAIRM1 is a prognostic biomarker and potential therapeutic target in glioma.

## INTRODUCTION

Glioma is the most prevalent and lethal primary brain tumor, accounting for nearly 30% of all cases [1]. Patients with glioma—especially glioblastoma multiforme (GBM)—have poor prognosis, with a median survival of approximately 14 months, even with treatment (surgery, radiotherapy, and adjuvant chemotherapy) [2, 3]. Several studies have examined the molecular mechanisms underlying glioma malignancy and identified promising therapeutic targets [3, 4]; however, the possibility of predicting prognosis and treatment effects in glioma is limited.

Long non-coding (lnc)RNAs, which are ncRNAs that are longer than 200 bases in length [5], are frequently found to be dysregulated in tumors. The lncRNA homeobox (HOX)A transcript antisense RNA (HOTAIR) located between the HOXC11 and HOXC12 loci is marker for glioma and other solid tumors [6]. HOTAIR promotes tumor progression by acting as a micro (mi)RNA sponge [7] and promoting an immunosuppressive tumor microenvironment [8].

HOTAIR myeloid-specific (HOTAIRM)1 is a lncRNA located between the HOXA1 and HOXA2 loci that was first identified as a myeloid-specific regulator of the HOXA gene family, which controls target gene transcription through chromosome remodeling during myeloid cell differentiation and maturation [9]. HOTAIRM1 functions as a tumor suppressor in colorectal, head and neck, gastric, and lung cancers by sponging micro (mi)RNAs and regulating immunosuppressive myeloid-derived suppressor cells [10–13]. However, HOTAIRM1 was also shown to promote cell proliferation and migration in pancreatic ductal adenocarcinoma [14]. Therefore, additional studies are needed to clarify the role of HOTAIRM1 in cancer.

To this end, in the present study we investigated the clinical relevance of HOTAIRM1 overexpression by analyzing 946 glioma specimens in The Cancer Genome Atlas (TCGA) and the Chinese Glioma Genome Atlas (CGGA) databases. We also examined the function of HOTAIRM1 by gene silencing in glioma cell lines. The results indicate that HOTAIRM1 plays an important role in the malignancy of glioma and is a potential therapeutic target for its treatment.

## RESULTS

### Association between HOTAIRM1 expression and clinical and molecular features of glioma

**Figure 1:**
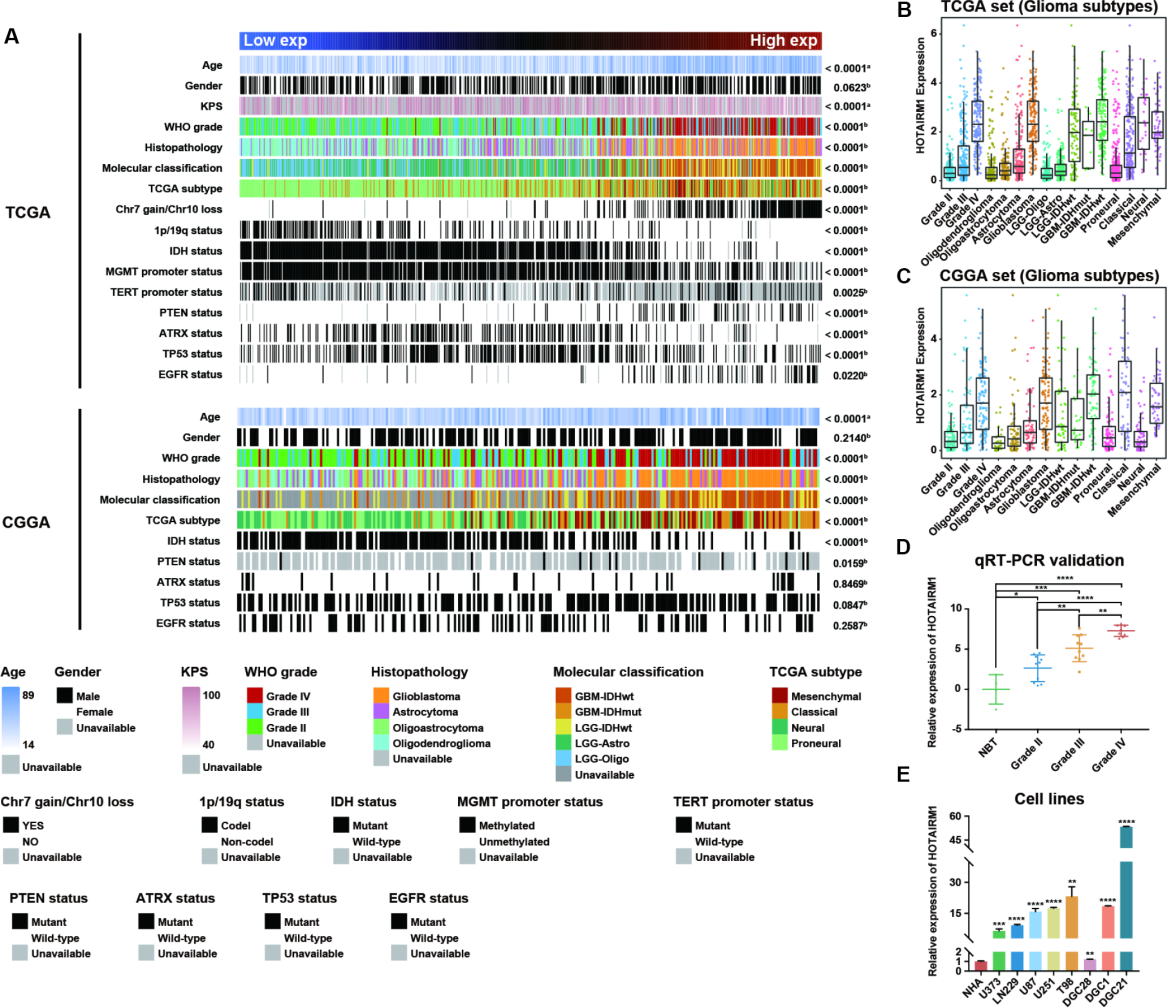
**Association between HOTAIRM1 expression and clinical and molecular features and malignancy in glioma.** (**A**) TCGA (top) and CGGA (bottom) data were arranged in ascending order of HOTAIRM1 expression level; the relationship between HOTAIRM1 level and clinical and molecular features of glioma was evaluated. a, Difference in continuous variables between high- and low-exp groups was assessed with the Student’s t test; b, distribution of categorical variables between high- and low-exp groups was assessed with the χ^2^ test or Fisher’s exact test. (**B**, **C**) HOTAIRM1 expression level according to tumor grade, histopathologic classification, and molecular and TCGA subtypes in TCGA (B) and CGGA (**C**). (**D**, **E**) qRT-PCR analysis of relative HOTAIRM1 expression level in four non-neoplastic brain tissue samples, 28 glioma tissue samples (Grade II, n = 10, Grade III, n = 10, and Grade IV, n = 8), normal human astrocytes (NHA), five glioma cell lines, and three primary DGC lines derived from glioma patients. Values represent mean ± SD. *P < 0.05, **P < 0.01, ***P < 0.001, ****P < 0.0001 (Student’s t test).

Samples from the TCGA and CGGA datasets were arranged according to increasing HOTAIRM1 expression level and the association between HOTAIRM1 expression and clinical and molecular features of glioma was examined (Figure 1A). Age at diagnosis was positively correlated with HOTAIRM1 level (Figure 1A and Supplementary Figure 1A, 1J). High HOTAIRM1 expression was frequently observed in patients with low Karnofsky Performance Score (KPS; Supplementary Figure 1A); these patients were more likely to exhibit a malignant phenotype (Figure 1A) and harbor wild-type alpha thalassemia/mental retardation syndrome X-linked (ATRX), isocitrate dehydrogenase (IDH), and telomerase reverse transcriptase promoter; mutant phosphatase and tensin homolog (PTEN), tumor protein (TP)53, and epidermal growth factor receptor (EGFR); and exhibit O-6-methylguanine-DNA methyltransferase (MGMT) promoter demethylation,1p/19q non-codeletion, and Chr7 gain/Chr10 loss (Figure 1A and Supplementary Figure 1A–1I and 1K–1O).

We compared clinical and molecular features between patients with high and low HOTAIRM1 expression and found that World Health Organization (WHO) grade, histopathology, IDH mutation status, MGMT promoter methylation status, and 1p/19q co-deletion status were associated with HOTAIRM1 level (Supplementary Table 3). Thus, HOTAIRM1 expression is correlated with clinical and molecular features of glioma.

### HOTAIRM1 is positively associated with glioma malignancy

To determine the relationship between HOTAIRM1 expression and glioma malignancy, we analyzed the association between HOTAIRM1 level and WHO grade and molecular classification. HOTAIRM1 overexpression was positively correlated with grade progression in TCGA specimens (Figure 1B). Of the five previously established molecular classifications [15], higher HOTAIRM1 expression was more frequently detected in low-grade glioma (LGG) with wild-type IDH (LGG-IDHwt) and GBM with wild-type IDH cases (Figure 1B). In terms of TCGA molecular subtypes, HOTAIRM1 expression was higher in the mesenchymal subtype than in the proneural subtype (Figure 1B and Supplementary Table 4). Analysis of the CGGA dataset yielded similar findings (Figure 1C and Supplementary Table 4).

We next examined HOTAIRM1 expression in an independent glioma cohort (Grade II, n = 10, Grade III, n = 10, and Grade IV, n = 8), four non-neoplastic brain tissue specimens, five glioma cell lines, one normal astrocyte cell line, and three primary glioma cell lines [differentiated glioblastoma cells (DGCs)] derived from glioma patients. Consistent with the above results, high HOTAIRM1 expression was associated with higher malignancy in these samples (Figure 1D, 1E), indicating that HOTAIRM1 overexpression is linked to glioma malignancy.

### High HOTAIRM1 expression predicts reduced survival of glioma patients

**Figure 2:**
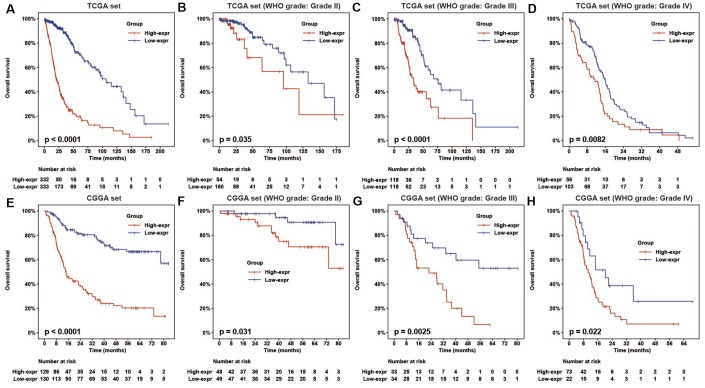
**HOTAIRM1 expression is correlated with glioma patient prognosis.** (**A**–**H**) Prognostic value of HOTAIRM1 in all cases (**A**, **E**) and according to glioma grade (**B**–**D**, **F**–**H**).

To determine the association between HOTAIRM1 expression and overall survival (OS) of glioma patients, we dichotomized median HOTAIRM1 levels into high expression (high-exp) and low expression (low-exp) groups. OS was shorter in the high-exp group compared to the low-exp group (median OS = 21.3 vs. 106.7 months, P < 0.0001; Figure 2A). We also examined the prognostic value of HOTAIRM1 in stratified groups (Grades II, III, and IV) and observed similar trends in the Kaplan–Meier survival curves (Figure 2A–2D). These results were confirmed in the CGGA dataset (Figure 2E–2H).

**Figure 3:**
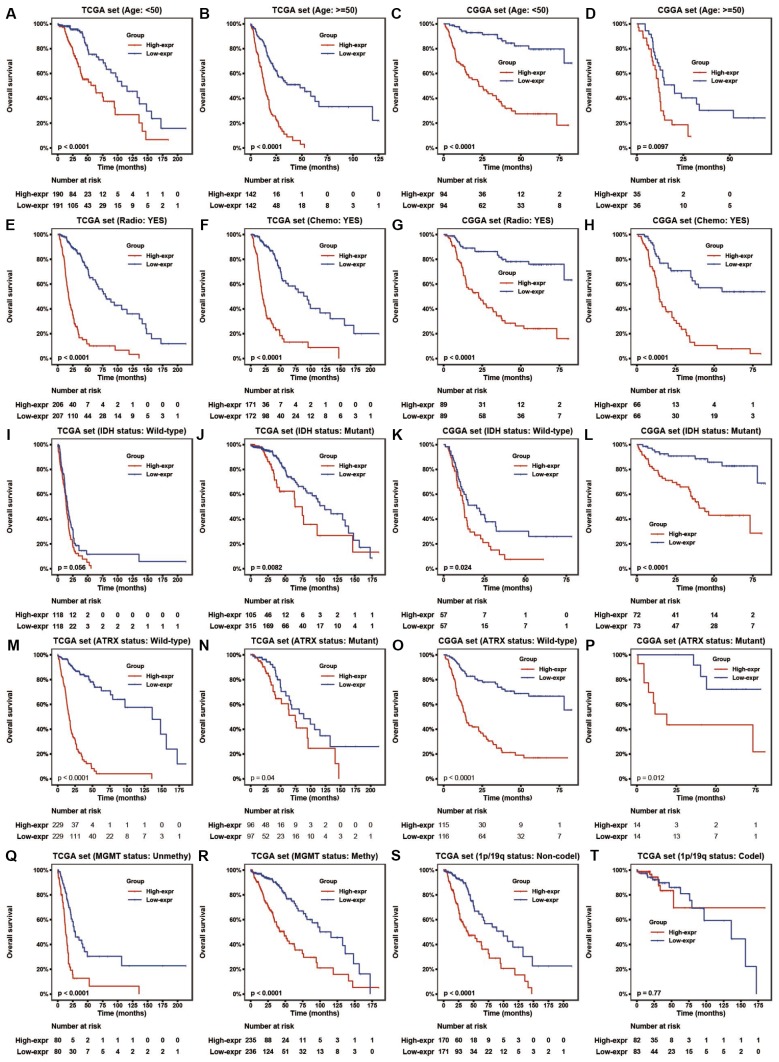
**HOTAIRM1 maintains high prognostic value in stratified groups.** (**A**–**T**) Prognostic value of HOTAIRM1 in TCGA and CGGA cohorts stratified by clinical (**A**–**H**) and genomic (**I**–**T**) features.

To assess the prognostic value of HOTAIRM1 in the stratified cohorts, patients were classified according to clinical factors (age, KPS, radiation, and chemotherapy) and genomic aberrations (IDH and ATRX mutation, MGMT promoter methylation, and 1p/19q co-deletion status). In most cohorts, high-exp patients had significantly shorter OS than those in the low-exp group (Figure 3A–3T and Supplementary Figure 2A, 2B) and tended to have poor prognosis, although the results were non-significant. We also examined the independence of HOTAIRM1 in the two datasets by Cox regression analysis. Uni- and multivariate analyses suggested that HOTAIRM1 is an independent prognostic factor in glioma [hazard ratio (HR) = 2.0559, P = 0.0042 in TCGA; HR = 2.6894, P < 0.0001 in CGGA; Table 1].

**Table 1:** Uni- and multivariate Cox analyses of prognostic factors in glioma.

**Variable**	**TCGA set**							**CGGA set**					
	**Number of samples**	**Univariate**			**Multivariate**			**Number of samples**	**Univariate**			**Multivariate**	
		**p value**	**HR**		**p value**	**HR**			**p value**	**HR**		**p value**	**HR**
**Age**													
Increasing years	670	<0.0001	1.0672		<0.0001	1.0405		274	<0.0001	1.0391		0.4957	0.9927
**Gender**													
Female vs Male	670	0.1456	0.8271					274	0.2944	0.8165			
**WHO Grade**													
Grade IV	160	ref.	ref.		ref.	ref.		101	ref.	ref.		ref.	ref.
Grade III	237	<0.0001	0.1496		0.0047	0.5426		72	<0.0001	0.3752		0.1054	0.6731
Grade II	216	<0.0001	0.1422		<0.0001	0.2554		101	<0.0001	0.0696		<0.0001	0.1109
**Radiotherapy**													
Yes vs No	611	0.0001	1.9199		0.0120	0.5843		255	0.0002	0.4646		0.0001	0.4274
**Chemotherapy**													
Yes vs No	396	0.0008	0.5529					248	0.0079	1.6935		0.0502	0.6420
**Chr7 gain/Chr10 loss**													
YES vs NO	663	<0.0001	8.1843		0.3358	1.2242		Unavailable	Unavailable	Unavailable		Unavailable	Unavailable
**1p/19q status**													
Codel vs Noncodel	666	<0.0001	0.2199		0.0803	0.5071		Unavailable	Unavailable	Unavailable		Unavailable	Unavailable
**IDH status**													
Mutation vs Wild-type	663	<0.0001	0.0993		0.0664	0.5265		274	<0.0001	0.2353		0.0854	0.6269
**ATRX status**													
Mutation vs Wild-type	658	<0.0001	0.4201		0.9901	0.9961		274	0.2168	0.6757			
**MGMT promoter status**													
Methylated vs Unmethylated	638	<0.0001	0.3022		0.1081	0.7417		Unavailable	Unavailable	Unavailable		Unavailable	Unavailable
**HOTAIRM1**													
High- vs Low-exp	672	<0.0001	5.8674		0.0042	2.0559		274	<0.0001	4.3906		0.0001	2.6894

The predictive value of HOTAIRM1 for 1-, 3-, and 5-year survival in TCGA and CGGA datasets was evaluated based on a receiver operating characteristic curve. The area under the curve for 1-, 3-, and 5-year survival was 74.56%, 75.87%, and 77.65%, respectively, in TCGA and 69.51%, 76.49%, and 76.89%, respectively, in CGGA. Thus, HOTAIRM1 is a better predictor of glioma patient survival than age, tumor grade, IDH mutation status, chemotherapy, and radiotherapy (Supplementary Figure 3A–3F).

### Functional analysis of HOTAIRM1 in glioma

**Figure 4:**
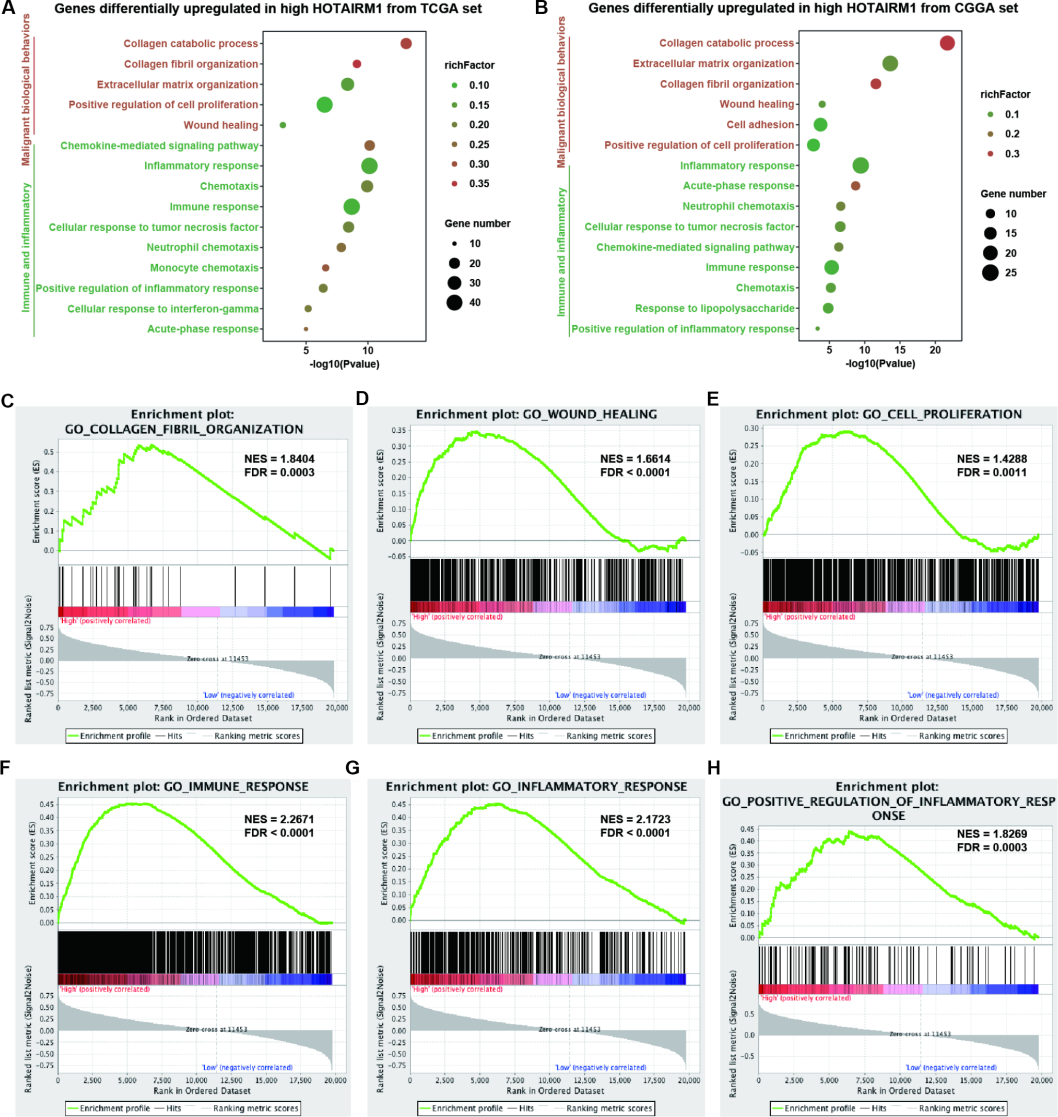
**Analysis of HOTAIRM1 function in glioma.** (**A**, **B**) GO analysis by DAVID showing biological processes associated with HOTAIRM1 in TCGA (**A**) and CGGA (**B**) datasets. (**C**–**H**) GSEA analysis was performed to verify the biological functions attributed to HOTAIRM1 in TCGA.

Gene expression profiles were analyzed to examine the phenotypes associated with HOTAIRM1 overexpression. TCGA data were used as the discovery set and CGGA data were used for validation in the whole-transcriptome principal components analysis (PCA). We found that patients with high and low HOTAIRM1 expression had distinct transcriptome profiles (Supplementary Figure 4A, 4B). Differentially expressed genes (DEGs) that were significantly upregulated (up-DEGs) in the high-exp group (log2 fold change > 2, P < 0.05; Supplementary Dataset 1) were associated with malignant tumor behavior and immune and inflammatory responses—e.g., collagen catabolism, wound healing, positive regulation of cell proliferation, cell adhesion, inflammatory response, cellular response to tumor necrosis factor, chemokine-mediated signaling, neutrophil chemotaxis, monocyte chemotaxis, response to lipopolysaccharide, acute-phase response, and immune response (Figure 4A, 4B). These results were validated by gene set enrichment analysis (GSEA) (Figure 4C–4H and Supplementary Figure 4C–4N).

### HOTAIRM1 promotes glioma proliferation and invasion in vitro

**Figure 5:**
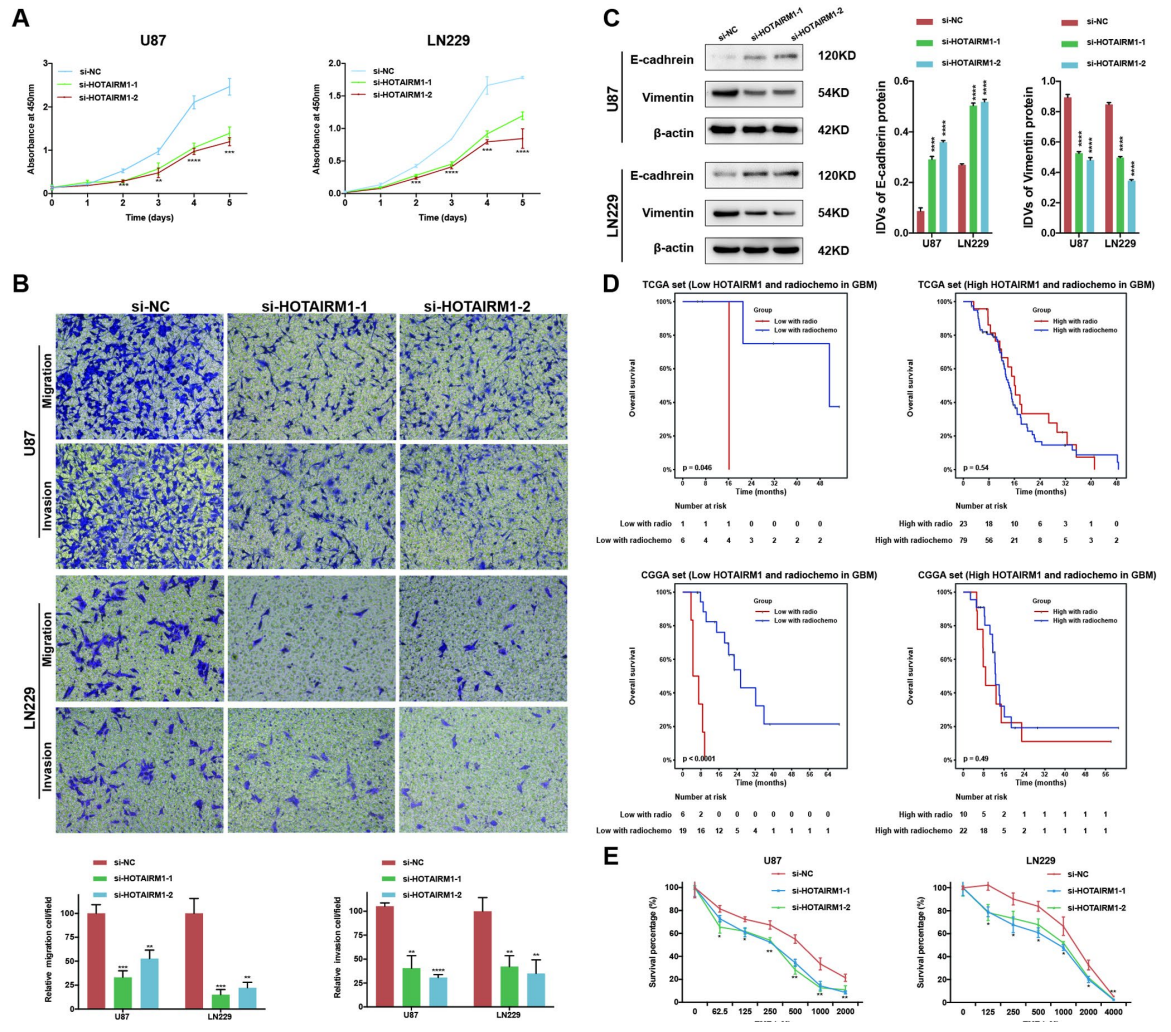
**HOTAIRM1 silencing inhibits glioma cell proliferation, migration, invasion, and EMT and increases sensitivity to TMZ in vitro.** (**A**) Cell proliferation after transfection of cells with si-HOTAIRM1-1 and -2, as determined with the CCK-8 assay. (**B**) Migration and invasion in U87 and LN229 cells evaluated with transwell assays. (**C**) Western blot analysis of E-cadherin (epithelial marker) and vimentin (mesenchymal marker) expression following HOTAIRM1 knockdown; β-actin served as a loading control. (**D**) Prognostic value of radiochemotherapy compared with radiotherapy alone in high- and low-exp groups. (**E**) Viability of U87 and LN229 cells transfected with siRNA against HOTAIRM1 (si-HOTAIRM1) or negative control siRNA (si-NC) following TMZ treatment at indicated doses. Values represent mean ± SD (n = 3 biological replicates). *P < 0.05, **P < 0.01, ***P < 0.001, ****P < 0.0001 (Student’s t test).

Small interfering (si)RNA-mediated knockdown of HOTAIRM1 expression in the U87 and LN229 glioma cell lines decreased HOTAIRM1 transcript level 48 h later (Supplementary Figure 5A). The results of the Cell Counting Kit (CCK)-8 assay showed that HOTAIRM1 silencing suppressed the proliferation of U87 and LN229 cells (Figure 5A), which was accompanied by decreased cell migration and invasion (Figure 5B and Supplementary Figure 5B). Thus, HOTAIRM1 has an oncogenic function in glioma.

### HOTAIRM1 promotes epithelial–mesenchymal transition (EMT) and temozolomide (TMZ) resistance

We performed PCA to investigate the functional significance of HOTAIRM1 overexpression in glioma. The results showed that high- and low-exp groups expressed distinct EMT gene sets, which was confirmed by GSEA (Supplementary Figure 6A, 6B). HOTAIMR1 knockdown increased E-cadherin and decreased vimentin protein levels relative to the control group (Figure 5C) and after 10 days, altered the morphology of U87 cells to a cobblestone-like appearance suggestive of mesenchymal-to-epithelial transition (Supplementary Figure 6C).

Given that EMT is associated with drug resistance, we explored the relationship between HOTAIRM1 expression and patient prognosis under different treatments. In patients who received radio- or chemotherapy, elevated HOTAIRM1 level was an indicator of poor prognosis (Figure 3E–3H). Additionally, patients in the low-exp group who received radiochemotherapy had longer OS than those treated with radiation alone, but this was not observed in patients with high HOTAIRM1 expression (Figure 5D). The results of the chemo-sensitivity assay showed that HOTAIRM1 knockdown in U87 and LN229 cells increased sensitivity to TMZ treatment, as evidenced by a 2-fold decrease in the half-maximal inhibitory concentration (IC50) of TMZ (Supplementary Table 5).

### Establishment of a HOTAIRM1–miRNA–mRNA network

**Figure 6:**
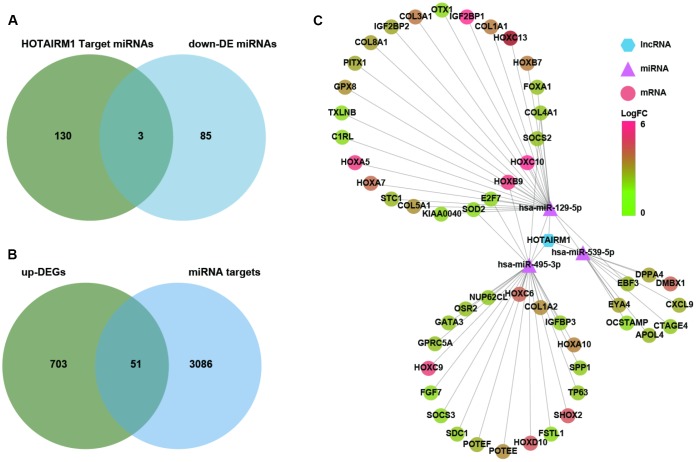
**Construction of a HOTAIRM1–miRNA–mRNA network.** (**A**) Venn diagram showing the intersection between HOTAIRM1 target miRNAs predicted with DIANA tools and LncBase Predicted v.2 and miRNAs that are differentially downregulated in GBM compared with normal brain tissue (P < 0.01). (**B**) Venn diagram showing the intersection between up-DEGs in the high-exp group compared with the low-exp group (log2 fold change > 2; P < 0.05) and predicted targets of three miRNAs (hsa-miR-129-5p, hsa-miR-495-3p, and hsa-miR-539-5p). (**C**) Network comprising HOTAIRM1, three miRNAs, and 51 genes generated with Cytoscape v.3.6.1. DE, differential expression.

Several lncRNAs act as a competing endogenous (ce)RNAs for miRNAs [16]. To investigate whether HOTAIRM1 has this function, putative target miRNAs of HOTAIRM1 in an online database were identified using DIANA tools and LncBase Predicted v.2. Three miRNAs that were downregulated in GBM compared with normal brain tissue (P < 0.01; Supplementary Dataset 2)—i.e., hsa-miR-129-5p, hsa-miR-495-3p, and hsa-miR-539-5p—were selected for further analysis (Figure 6A). We identified 3137 target genes of these three miRNAs in starBase v.3.0. Genes in the overlap between miRNA targets and up-DEGs were screened (754 genes; Supplementary Dataset 1), yielding a set of 51 genes for further analysis (Figure 6B).

**Figure 7:**
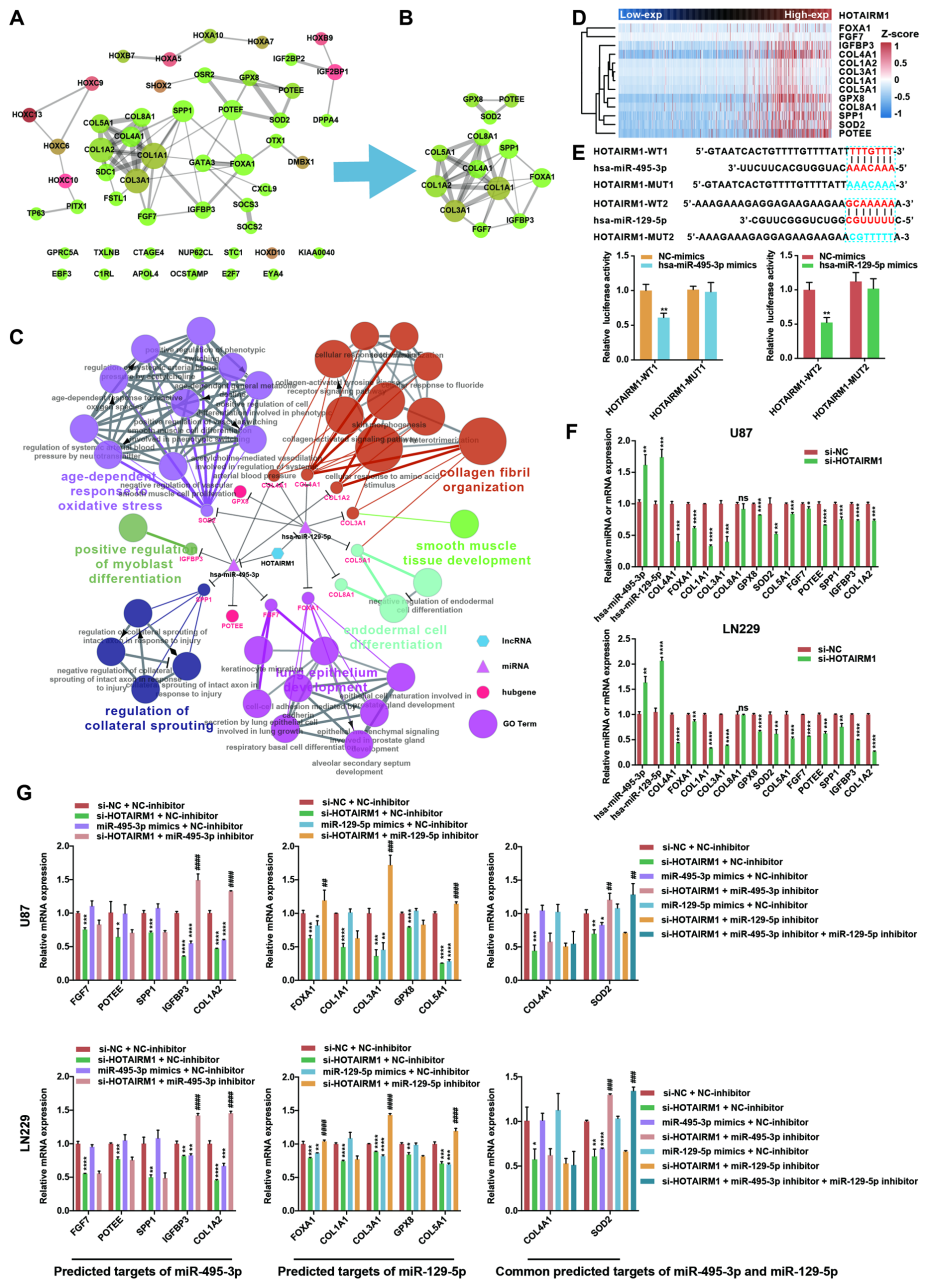
**Construction of a HOTAIRM1–miRNA–hub gene network.** (**A**) PPI network of 51 target genes that are closely related to HOTAIRM1 level generated using the PPI function in Cytoscape v.3.6.1. Node size increased according to the number of neighbors of each gene, and edge size changed from fine to coarse based on the combined score of two adjacent genes. (**B**) Subnetwork consisting of 13 hub genes extracted from (**A**) generated by MCODE in Cytoscape v.3.6.1. (**C**) Network consisting of HOTAIRM1, two miRNAs (hsa-miR-129-5p and hsa-miR-495-3p), and 13 hub genes generated with Cytoscape v.3.6.1. GO analyses of 13 hub genes was performed using ClueGO. (**D**) Expression profile of 13 hub genes in order of increasing HOTAIRM1 expression level in TCGA dataset. (**E**) Schematic representation of putative binding sites of miR-495-3p and miR-129-5p in HOTAIRM1. Relative luciferase activity was determined for HEK-293T cells co-transfected with wild-type HOTAIRM1 overexpression plasmid (HOTAIRM1-WT1) and miR-459-3p mimic or with HOTAIRM1-WT2 and miR-129-5p mimic. **P < 0.01 vs. HOTAIRM1-WT1 + negative control (NC) mimic or HOTAIRM1-WT2 + NC mimic group. (**F**) qRT-PCR analysis of expression levels of miR-129-5p, miR-495-3p, and 13 hub genes after HOTAIRM1 knockdown. ns, no significant difference; *P < 0.05, **P < 0.01, ***P < 0.001, ****P < 0.0001 vs. negative control siRNA (si-NC) group. (**G**) qRT-PCR analysis of the levels of indicated hub genes in U87 and LN229 cells after HOTAIRM1 knockdown and transfection of indicated miRNA inhibitor or mimic. *P < 0.05, **P < 0.01, ***P < 0.001, ****P < 0.0001 vs. si-NC + NC inhibitor group; ^##^P < 0.01, ^###^P < 0.001, ^####^P < 0.0001 vs. si-HOTAIRM1 + NC inhibitor group.

We constructed a lncRNA–miRNA–mRNA (i.e., ceRNA) network (Figure 6C) to gain insight into the relationship between HOTAIRM1, its target miRNAs, and up-DEGs. A protein–protein interaction (PPI) network was developed to evaluate interactions among the 51 target genes (Figure 7A). The Molecular Complex Detection (MCODE) approach was used to identify hub genes from the PPI network. Using k-core = 2 and degree cutoff = 3, we identified a sub-network containing 13 nodes and 31 edges (Figure 7B). A HOTAIRM1–miRNA–hub gene network was established to determine the association between HOTAIRM1, miRNAs, and hub genes (Figure 7C). The network had 15 lncRNA–miRNA–mRNA regulatory axes comprising HOTAIRM1, two miRNAs (hsa-miR-129-5p and hsa-miR-495-3p), and 13 hub genes. Gene Ontology (GO) analysis of the hub genes using ClueGO showed that most of the biological functions were enriched in cytoskeleton and cell differentiation, which play an important role in cell migration and invasion (Figure 7C). We also analyzed the hsa-miR-129-5p and hsa-miR-495-3p binding sites in HOTAIRM1 and hub gene transcripts (Figure 7E and Supplementary Figure 7) and determined with a luciferase reporter assay that both hsa-miR-129-5p and hsa-miR-495-3p directly bind to HOTAIRM1 (Figure 7E); furthermore, both were upregulated by HOTAIRM1 knockdown (Figure 7F). Conversely, suppression of HOTAIRM1 decreased the expression of 12 of the 13 hub genes that were upregulated in the high-exp group (Figure 7D, 7F). However, in HOTAIRM1-depleted U87 and LN229 cells, only insulin-like growth factor-binding protein (IGFBP)3, collagen type I α2 chain, and superoxide dismutase (SOD)2 levels were restored by hsa-miR-495-3p inhibition whereas Forkhead box A1, collagen type III α1 chain, and collagen type V α1 chain levels were restored by inhibiting hsa-miR-129-5p. These data suggest that HOTAIRM1 functions as a ceRNA to promote glioma cell migration and invasion.

### High HOTAIRM1 expression is associated with a distinct immune and inflammatory phenotype

**Figure 8:**
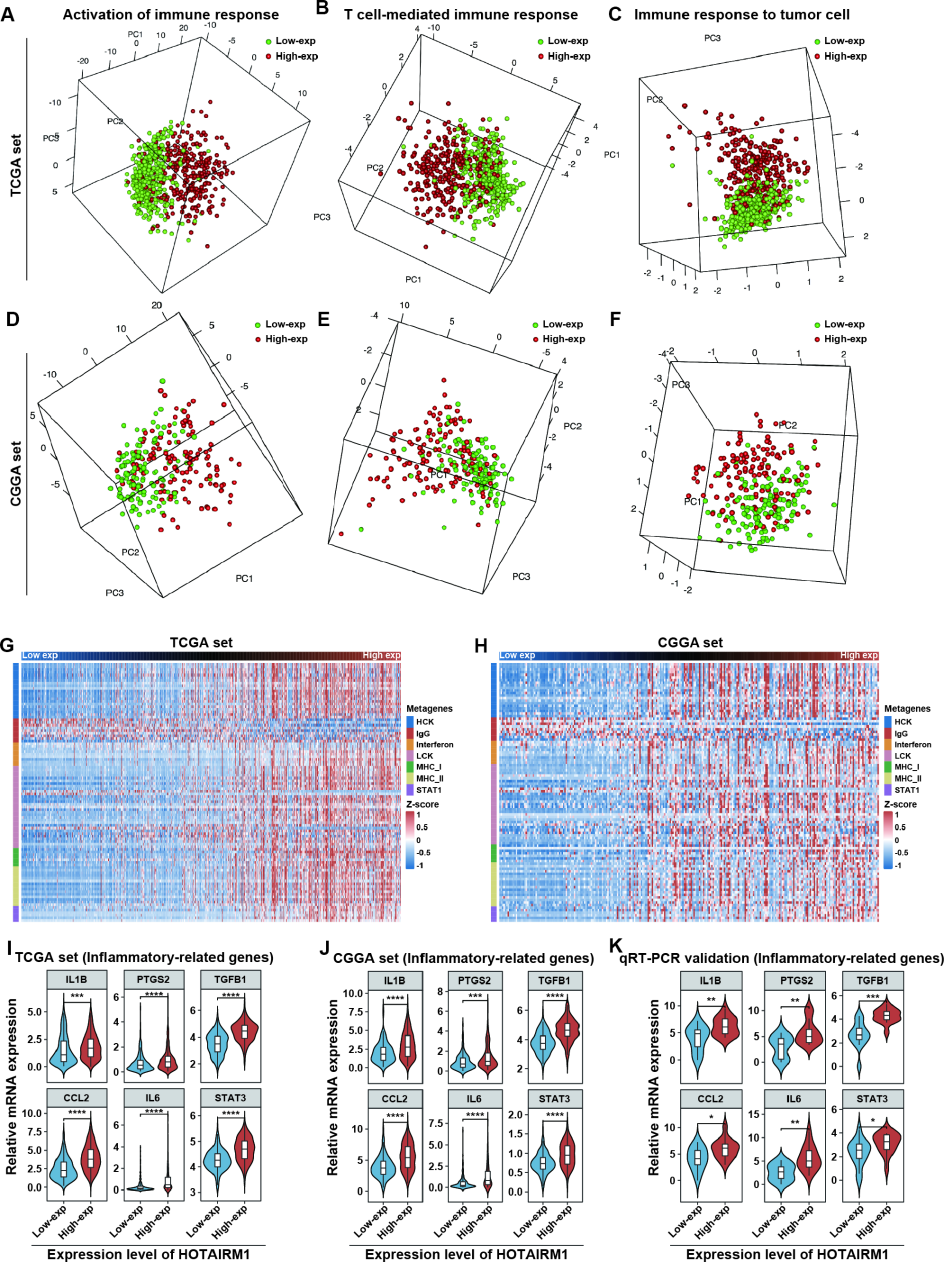
**Elevated HOTAIRM1 expression is associated with distinct immune and inflammatory phenotypes.** (**A**–**F**) Activation of T cell-mediated immune response and immune response to tumor cells is associated with HOTAIRM1 expression levels in TCGA (**A**–**C**) and CGGA (**D**–**F**). (**G**, **H**) Effect of HOTAIRM1 on inflammation in TCGA (**G**) and CGGA (**H**); gene expression level was normalized to z score. (**I**–**K**) Expression of six inflammation-related genes in TCGA (**I**), CGGA (**J**), and independent glioma samples (**K**) in relation to HOTAIRM1 level. *P < 0.05, **P < 0.01, ***P < 0.001, ****P < 0.0001 vs. low-exp group (Student’s t test).

GO analyses revealed that immune response and inflammatory activity in glioma were influenced by HOTAIRM1 level (Figure 4A, 4B). Some of the above-mentioned hub genes, including secreted phosphoprotein (SPP)1, IGFBP3, SOD2, and fibroblast growth factor 7 are involved in the regulation of the tumor immune response [17–20]. We examined the relationship between HOTAIRM1 and local immune status by performing PCA of gene sets related to immune activation, T cell-mediated immune response, and immune response to tumor cells extracted from Molecular Signatures Database v.6.2 (http://software.broadinstitute.org/gsea/index.jsp; Supplementary Dataset 3). The results showed that the pattern of immune responses varied according to HOTAIRM1 expression level, which was confirmed by GSEA (Figure 8A–8F and Supplementary Figure 8). An analysis of inflammatory response [21, 22] according to the expression level of HOTAIRM1 revealed that most inflammation-related genes were positively associated with HOTAIRM1 expression except for immunoglobulin G, which was related to B lymphocyte activity (Figure 8G, 8H and Supplementary Figure 9). We then compared the expression levels of six inflammation-related genes including interleukin 1 beta, prostaglandin-endoperoxide synthase 2 (also known as cyclooxygenase 2b), transforming growth factor beta 1, C-C motif chemokine ligand 2, interleukin 6, and signal transducer and activator of transcription 3 [23, 24] between high- and low-exp groups in TCGA and CGGA. All six of these genes were more highly expressed in the high-exp group than in the low-exp group, which was confirmed by quantitative real-time polymerase chain reaction (qRT-PCR) in an independent glioma cohort (Figure 8I–8K). Thus, aberrant HOTAIRM1 expression is associated with a distinct immune and inflammatory phenotype in glioma.

To evaluate the impact of HOTAIRM1 on the tumor microenvironment (TME), we calculated immune and stromal scores and tumor purity in glioma specimens of TCGA and CGGA datasets. HOTAIRM1 expression was positively associated with immune and stromal scores and negatively associated with tumor purity. To determine which type of non-tumor cell contributes to the altered TME, we used gene set variation analysis (GSVA) to estimate the gene set of immune cells and evaluate the correlation between immune cell types and HOTAIRM1 expression. We used a threshold of absolute correlation coefficient > 0.2 and false discovery rate < 0.01 in the two datasets. The results showed that delta T cells, cluster of differentiation 8+ T cells, activated dendritic cells, and regulatory T cells (Tregs) were positively correlated, whereas follicular B helper T cells were negatively correlated with HOTAIRM1 expression (Supplementary Figure 10).

## DISCUSSION

LncRNAs are implicated in tumorigenesis and immune system regulation [25] and modulate various aspects of glioma biology including proliferation, migration, invasion, and drug resistance [7, 26]. In this study we found a close association between HOTAIRM1 expression and the clinical and molecular characteristics of glioma. Elevated HOTAIRM1 was linked to high risk factors including older age (≥ 50 years), lower KPS, mesenchymal subtype, wild-type IDH, unmethylated MGMT promoter, and 1p/19q non-codeletion, whereas protective factors such as wild-type PTEN and TP53 were associated with glioma samples showing low HOTAIRM1 expression. These findings indicate that HOTAIRM1 has an oncogenic function in human glioma.

HOTAIRM1 has been shown to function as both an oncogene and tumor suppressor in various solid tumors. For instance, HOTAIRM1 inhibits tumorigenesis by forming ceRNA networks in colorectal cancer, head and neck tumors, gastric cancer, and hepatocellular carcinoma [10–12, 27]. Conversely, HOTAIRM1 promotes breast cancer, lung cancer, and pancreatic ductal adenocarcinoma by directly regulating HOXA1 expression [13, 14, 28, 29]. As a fetal lncRNA [30], HOTAIRM1 is upregulated in glioma [31, 32]. However, the prognostic value of HOTAIRM1 in glioma remains unknown. To address this point, we analyzed TCGA and CGGA cohorts and found that HOTAIRM1 upregulation was independently associated with poor prognosis in glioma patients. Functional analyses revealed that HOTAIRM1 is involved in various malignant behaviors, which is consistent with a recent report [33]. Thus, HOTAIRM1 is an onco-lncRNA in glioma.

Resistance to alkylating agents is the main reason for the poor prognosis of glioma even after chemotherapy. EMT is a key driver of chemotherapy resistance, which is regulated by various lncRNAs. In glioma, HOXA transcript at the distal tip induces the expression of zinc finger E-box-binding homeobox 1 and promotes EMT by sponging miR-101 under hypoxic conditions [34]. In the present study, HOTAIRM1 knockdown inhibited EMT in glioma by reducing the levels of mesenchymal cell markers and increasing those of epithelial cell markers. HOTAIRM1 overexpression was associated with limited clinical benefit from TMZ treatment, whereas HOTAIRM1 knockdown decreased the IC50 for TMZ in two glioma cell lines. These results suggest that HOTAIRM1 can serve as a marker for TMZ resistance and that its inhibition is a useful therapeutic strategy for glioma patients.

HOTAIRM1 was shown to promote tumor malignancy by directly regulating HOXA1 expression in lung cancer and glioma [13, 33]. Consistent with these observations, we found that HOTAIRM1 level was positively associated with that of HOXA family genes in TCGA and CGGA datasets (Supplementary Figure 11). CeRNA networks formed in conjunction with miRNAs and target genes are an important mechanism by which lncRNAs regulate tumorigenesis [26]. We established a ceRNA network comprising HOTAIRM1, hsa-miR-129-5p, hsa-miR-495-3p, and 13 hub genes that was validated with the luciferase assay and by qRT-PCR. Previous studies have demonstrated that hsa-miR-129-5p and hsa-miR-495-3p inhibit cell proliferation, migration, invasion, and EMT and increase drug sensitivity [35–37]. Accordingly, most of the identified 13 hub genes were associated with tumor progression and establishment of the TME [17–19, 38–40]. For example, SPP1 (also known as osteopontin) is known to promote glioma progression by regulating GBM-associated macrophage infiltration [17], while IGFBP3 impairs T cell accumulation in breast cancer [41]. However, hsa-miR-129-5p and hsa-miR-495-3p inhibitors restored the expression of only a subset of hub genes following depletion of HOTAIRM1, indicating that the latter promotes malignancy and regulates the TME in glioma not only by sponging hsa-miR-129-5p and hsa-miR-495-3p but also by enhancing the expression of some hub genes within a non-ceRNA.

We previously showed that different histological and molecular subtypes of glioma are characterized by distinct local immune status [15, 42]. There is accumulating evidence that lncRNAs regulate immune processes. For example, lincRNA-Cox2 and lincRNA-NeST are regulators of innate immunity [43, 44], and lnc-EGFR mediates immunosuppression in hepatocellular carcinoma by stimulating Treg differentiation [45]. Similarly, HOTAIRM1 was shown to promote an immunosuppressive state in the TME [13]. Here we showed that HOTAIRM1 expression was associated with activation of a T cell-mediated immune response and increased inflammation. HOTAIRM1 also enhanced the immunosuppressive microenvironment of glioma by recruiting Tregs and dendritic cells. Given that EMT is closely related to an immunosuppressive status [46], we speculate that HOTAIRM1 promotes EMT to reprogram immune and inflammatory responses within the glioma microenvironment.

The results of this study demonstrate that HOTAIRM1 can serve as an independent factor for predicting glioma patient survival and response to TMZ chemotherapy. A ceRNA network was established that explains the potential mechanism of action of HOTAIRM1. However, one limitation of the present study is that some of our conclusions were based on the result of a retrospective analysis of publicly available datasets. Thus, additional experiments are needed to clarify the biological function and molecular mechanisms of HOTAIRM1 in glioma.

## MATERIALS AND METHODS

### Patient samples

Glioma tissue samples (n = 28) for qRT-PCR were collected at the First Hospital of China Medical University. The histological diagnoses of the samples were independently verified by two experienced neuropathologists based on 2010 WHO classification guidelines. Four non-neoplastic brain tissue specimens from cranial injury internal decompression patients served as the negative control. The present study was approved by the Ethics Committee of the First Hospital of China Medical University.

### Glioma databases

RNA-seq data, including lncRNA expression in 672 glioma samples were acquired from TCGA database (http://cancergenome.nih.gov/). CGGA transcriptome sequencing data of 274 glioma samples were downloaded from the public database (http://www.cgga.org.cn). After obtaining the RNA-seq data, we annotated each of the samples according to their barcode ID based on available clinical information from University of California, Santa Cruz Xena (https://xenabrowser.net/datapages/) and CGGA databases. A total of 946 glioma samples from TCGA and CGGA with detailed clinical and molecular information were ultimately included in the analysis (Supplementary Table 1). miRNA expression data were downloaded from TCGA database.

### Cell lines

U87, LN229, U373, U251, and T98G human glioma cell lines and normal human astrocytes were purchased from American Type Culture Collection (Manassas, VA, USA) and cultured in Dulbecco’s Modified Eagle’s Medium (DMEM; HyClone, Logan, UT, USA) supplemented with fetal bovine serum (FBS) and penicillin/streptomycin (100 U/ml). DGCs were derived from freshly resected GBM patient specimens and cultured in Roswell Park Memorial Institute 1640 Medium Modified (HyClone) supplemented with FBS and penicillin/streptomycin (100 U/ml). Cells were cultured in an atmosphere of 5% CO_2_ at 37°C. All tumor samples were obtained according to the protocol approved by the Ethics Committee of the First Hospital of China Medical University.

### Cell transfection

siRNAs for HOTAIRM1 knockdown were synthesized by Sangon Biotech (Shanghai, China) and had the following sequences: si-HOTAIRM1-1 sense, 5′-AGAAACUCCGUGUUACUCATT-3′; si-HOTAIRM1-2 sense, 5′-GAUUAAUCAACCACACUGATT-3'; and si-HOTAIRM1-3 sense, 5′-CCAAUUUAAAUCUAUGGCUTT-3′. hsa-miR-495-3p and hsa-miR-129-5p mimic and inhibitor and a negative control (Sangon Biotech) were used in this study.

### qRT-PCR

Total RNA was extracted from samples using TRIzol reagent (Life Technologies, Carlsbad, CA, USA), and cDNA was synthesized using an RNA PCR kit (Takara Bio, Otsu, Japan). qRT-PCR was performed using TB Green Premix Ex Taq II (Tli RNaseH Plus) (Takara Bio), according to the manufacturer’s protocol, with the 18S gene sequence serving as the internal standard. To evaluate miR-129-5p and miR-495-3p levels, cDNA was generated with the Taqman miRNA Reverse Transcription kit (Applied Biosystems, Foster City, CA, USA) and qRT-PCR was performed with a TaqMan miRNA assay using TaqMan Universal Master Mix II (Applied Biosystems); the U6 gene served as an endogenous control. qRT-PCR was performed on a LightCycler 96 System (Roche Diagnostics, Basel, Switzerland). All reactions were performed in triplicate. Relative expression levels were calculated with the 2^−ΔΔCt^ method. The primer sequences and miRNA assay IDs are shown in Supplementary Table 2.

### Protein isolation and western blotting

Cells were collected, washed twice with phosphate-buffered saline, and incubated on ice for 20–30 min in radioimmunoprecipitation assay lysis buffer containing 1% benzene sulfonyl fluoride (Beyotime Institute of Biotechnology, Shanghai, China). Protein concentration was measured using a bicinchoninic acid protein assay kit (Beyotime Institute of Biotechnology), and proteins were separated by 6%–15% sodium dodecyl sulfate polyacrylamide gel electrophoresis (Beyotime Institute of Biotechnology), transferred to a polyvinylidene difluoride membrane (Millipore, Billerica, CA, USA), and incubated for 60 min in 5% non-fat milk followed by primary antibodies against E-cadherin (1:10,000; Abcam, Cambridge, UK) and vimentin (1:5000; Abcam). An antibody against β-actin (1:1000; Proteintech, Rosemont, IL, USA) was used as the loading control. The membrane was incubated for 2 h at room temperature with horseradish peroxidase-conjugated secondary antibodies and protein bands were detected by enhanced chemiluminescence. Relative density values were calculated using ImageJ software (National Institutes of Health, Bethesda, MD, USA).

### Cell proliferation, wound healing, migration, and invasion assays

Cell proliferation was assessed using CCK-8 (KeyGen Biotech, Nanjing, China). After transfection of HOTAIRM1 siRNA or a negative control siRNA for 48 h, 2 × 10^3^ cells per well were seeded in 96-well plates and cultured for 0, 1, 2, 3, 4, and 5 days. A 10-μl volume of CCK-8 solution was added to each well and incubated at 37°C for 1 h, and the absorbance at 450 nm was measured. All experiments were performed in triplicate.

For the wound healing assay, approximately 1 × 10^6^ cells per cell were seeded in 6-well plates and incubated at 37°C until they reached at least 90% confluence. Wounds were created by scratching cell monolayers with a 1000-μl plastic pipette tip, followed by incubation in fresh medium without serum for 24 h. The initial gap length (0 h) and gap length after 24 h were measured from photomicrographs.

Cell migration and invasion assays were performed using 24-well Transwell chambers with 8-μm pore size polycarbonate membranes (Corning Inc., Corning, NY, USA). For the cell migration assay, 2 × 10^4^ cells were seeded on the top side of the membrane without Matrigel (BD Biosciences, Franklin Lakes, NJ, USA) in serum-free DMEM. The lower chamber was filled with 600 μl DMEM containing 20% FBS. After incubation at 37°C for 20 h, cells migrating to the lower surface of the membrane were fixed and stained with 0.5% crystal violet. For the invasion assay, 6 × 10^4^ cells were seeded on the top side of the membrane pre-coated with Matrigel, and invaded cells on the lower membrane surface were fixed and stained after 24 h. Migration and invasion were quantified by counting cells in three random fields of view per well under 10× objective.

### *In vitro* chemosensitivity assay

TMZ was supplied by Tasly Pharmaceutical (Tianjin, China) and dissolved in dimethyl sulfoxide to a concentration of 100 mM and diluted in cell culture medium to the appropriate final concentration. Approximately 5 × 10^3^ cells per well were seeded in a 96-well plate and incubated at 37°C for 24 h. TMZ was added at concentrations ranging from 62.5 to 4000 μM. Cell viability was assessed with the CCK-8 kit. A dose–response curve was plotted, and IC_50_ values were calculated by non-linear regression (curve fit) with Prism v.7.0a software (GraphPad, La Jolla, CA, USA). Three independent experiments were performed.

### Construction of lncRNA–miRNA–mRNA network

miRNAs that were predicted to bind HOTAIRM1 were identified from DIANA tools and LncBase Predicted v.2 (http://www.microrna.gr/LncBase/). LncRNA–miRNA pairs were determined from the intersection of miRNAs that were predicted to bind HOTAIRM1 and those that were downregulated in GBM compared with normal brain tissue (P < 0.01). Target genes of overlapping miRNAs were predicted with starBase v.3.0 (http://starbase.sysu.edu.cn), which includes seven prediction algorithms (PITA, RNA22, miRmap, microT, miRanda, PicTar, and TargetScan). miRNA–mRNA pairs were identified by overlapping target genes predicted by at least three algorithms with up-DEGs in the high- vs. low-exp group. lncRNA–miRNA and miRNA–mRNA pairs were used to construct a lncRNA–miRNA–mRNA network using Cytoscape v.3.6.1 software.

### Dual luciferase reporter assay

The pmirGLO luciferase vector with putative hsa-miR-495-3p and hsa-miR-129-5p binding sites in HOTAIRM1 and their mutated forms were purchased from Promega (Madison, WI, USA). The plasmid and miRNA mimic were co-transfected into HEK-293T cells, which were lysed 48 h later and tested for firefly and Renilla luciferase activities using the Dual-Luciferase Reporter Assay System (Promega). Renilla luciferase activity served as an internal control.

### Bioinformatic analysis

PCA was performed using R package to evaluate genomic expression patterns in relation to HOTAIRM1 expression levels. GO analysis was performed using DAVID (http://david.abcc.ncifcrf.gov/home.jsp) or ClueGO, a Cytoscape plug. Immune score, stromal score, and glioma purity were calculated with the Estimation of Stromal and Immune Cells in Malignant Tumors Using Expression Data R package (https://sourceforge.net/projects/estimateproject/) as previously described [47]. The GSVA package was used to assess the relationship between HOTAIRM1 expression level and immune cell abundance/inflammation in gene sets retrieved from previous studies [21]. GSEA of biological functions was performed (http://software.broadinstitute.org/gsea/index.jsp), and a PPI network was constructed using the STRING protein query function in Cytoscape v.3.6.1. Hub genes in the PPI network were identified with MCODE [48], a Cytoscape v.3.6.1 plug-in.

### Statistical analysis

Statistical analyses were performed using Excel 2016 (Microsoft, Redmond, WA, USA), SPSS v.24 (SPSS Inc., Chicago, IL, USA), Prism 7 v.7.0a, or R v.3.5.0 (https://www.r-project.org/) software. Glioma patients were divided into a low- and a high-exp groups according to median expression level of HOTARIM1. Differences in clinical and molecular features between the two groups were evaluated with the Student’s t test or χ^2^ test, and differences in HOTAIRM1 expression levels between the two groups were evaluated with the Student’s t test or by one-way analysis of variance. A Kaplan–Meier survival curve was used to assess the prognostic significance of HOTAIRM1 expression. Uni- and multivariate Cox regression analyses were performed to identify independent prognostic factors for glioma. A two-sided P value < 0.05 was considered statistically significant.

## Supplementary Material

Supplementary Figures

Supplementary Tables

Supplementary Dataset 1

Supplementary Dataset 2

Supplementary Dataset 3
