# Supplementary material for: Long non-coding RNA, HOTAIRM1, promotes glioma malignancy by forming a ceRNA network
Source: Aging (Albany NY). 2019 Sep 2;11(17):6805–38. doi: 10.18632/aging.102205 (PMC6756894; doi:10.18632/aging.102205)
Supplement: Supplementary Figures [file aging-11-102205-s005.pdf]

SUPPLEMENTARY FIGURES

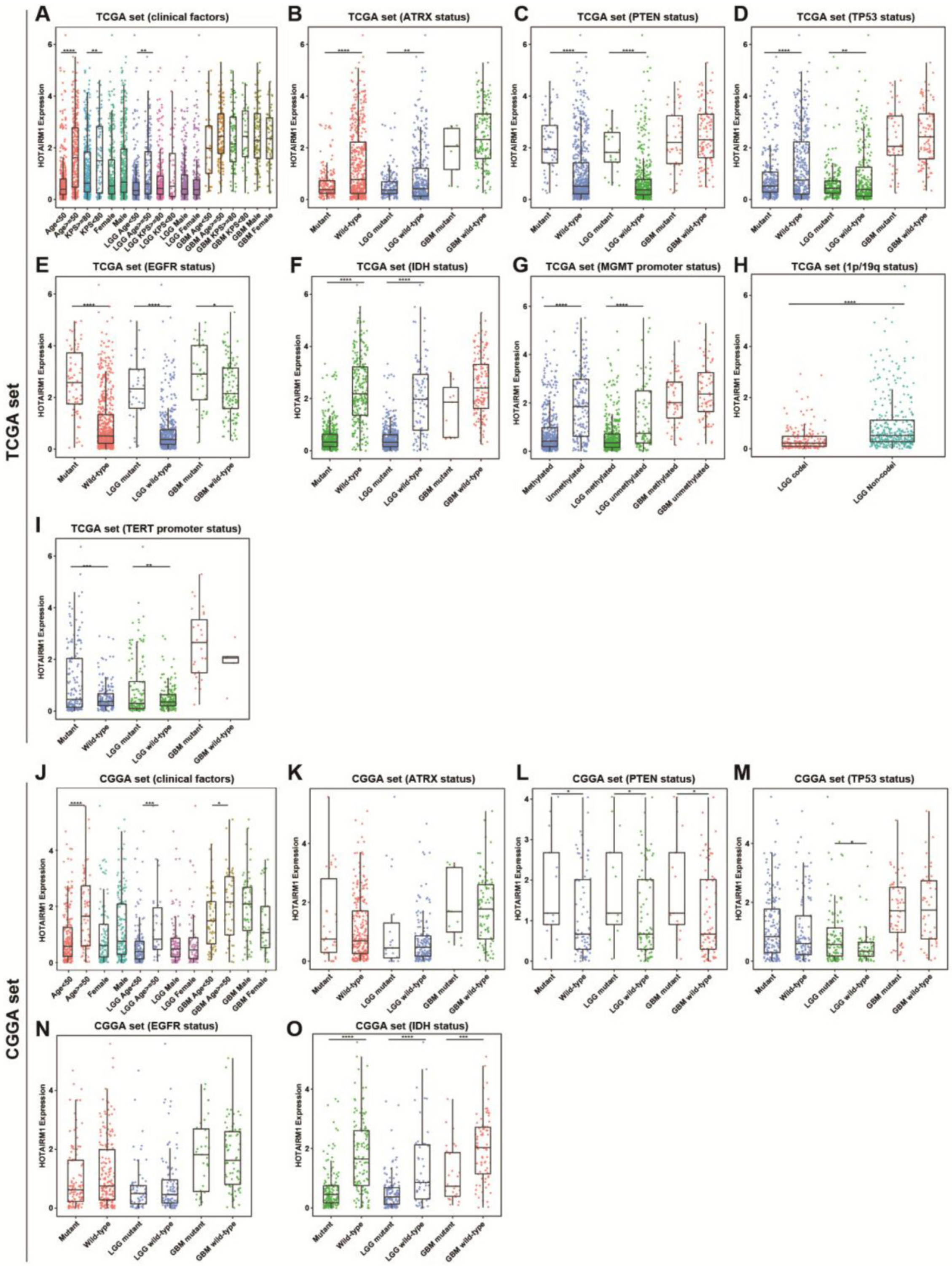

Supplementary Figure 1. Distribution of HOTAIRM1 expression level associated with clinical and molecular features. Distribution of HOTAIRM1 expression level in TCGA (**A**–**I**) and CGGA (**J**–**O**). ns, no significant difference, *p < 0.05, **p < 0.01, ***p < 0.001, ****p < 0.0001, values represented as the mean ± SD, Student's t-test.

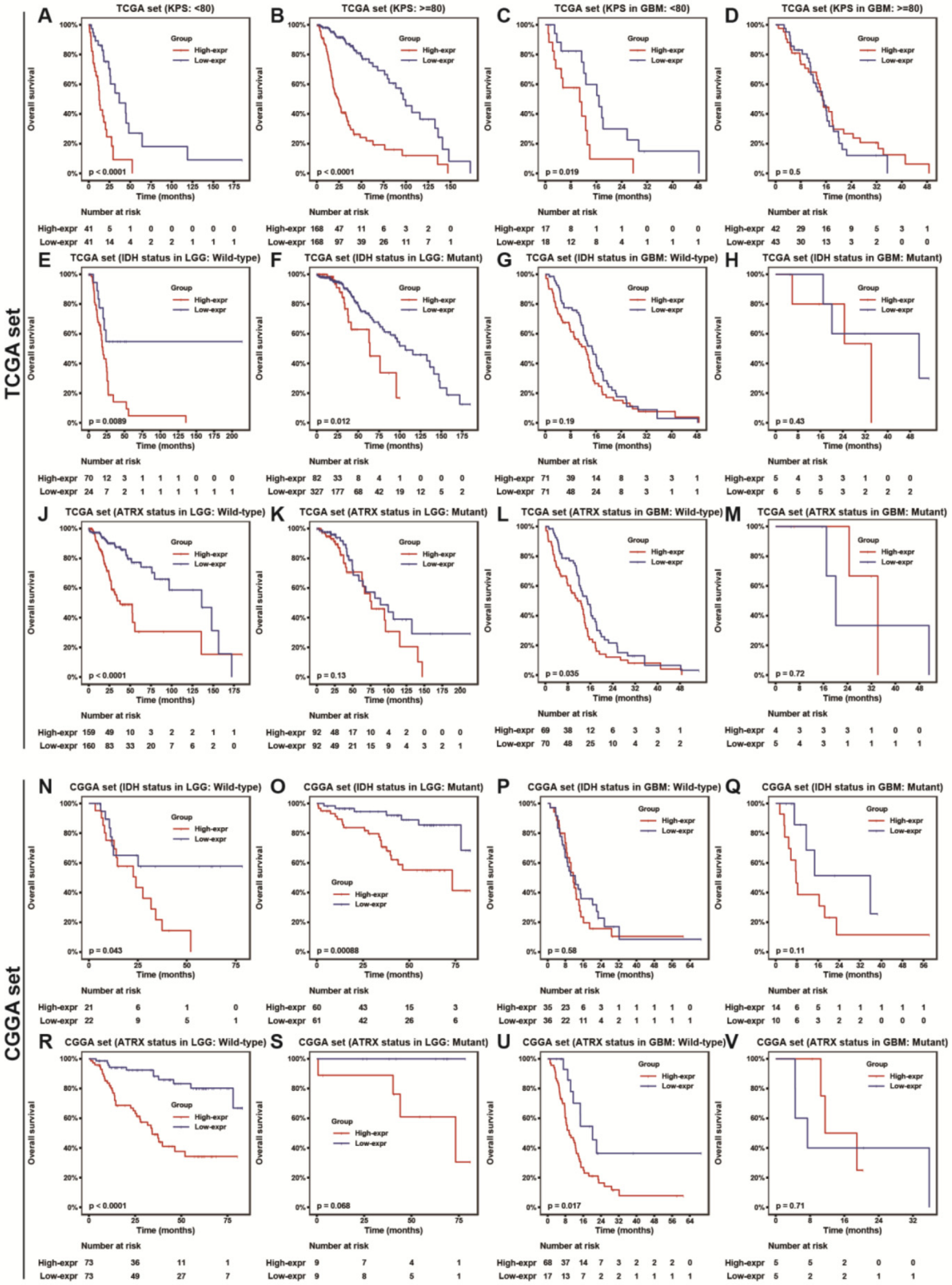

Supplementary Figure 2. Application of HOTAIRM1 in cohorts stratified by clinical and molecular features. (**A**–**D**) Prognostic value of HOTAIRM1 for the KPS in glioma or GBM in TCGA. HOTAIRM1 showed predictive value in LGG cohorts stratified by IDH status in TCGA (**E**, **F**) and CGGA (**N**, **O**) but not in GBM cohorts (**G**, **H**, **P**, **Q**). High HOTAIRM1 expression indicated a poor prognosis in LGG or GBM with ATRX-wt (**J**, **L**, **R**, **U**) but not in LGG or GBM with ATRX-mut (**K**, **M**, **S**, **V**)

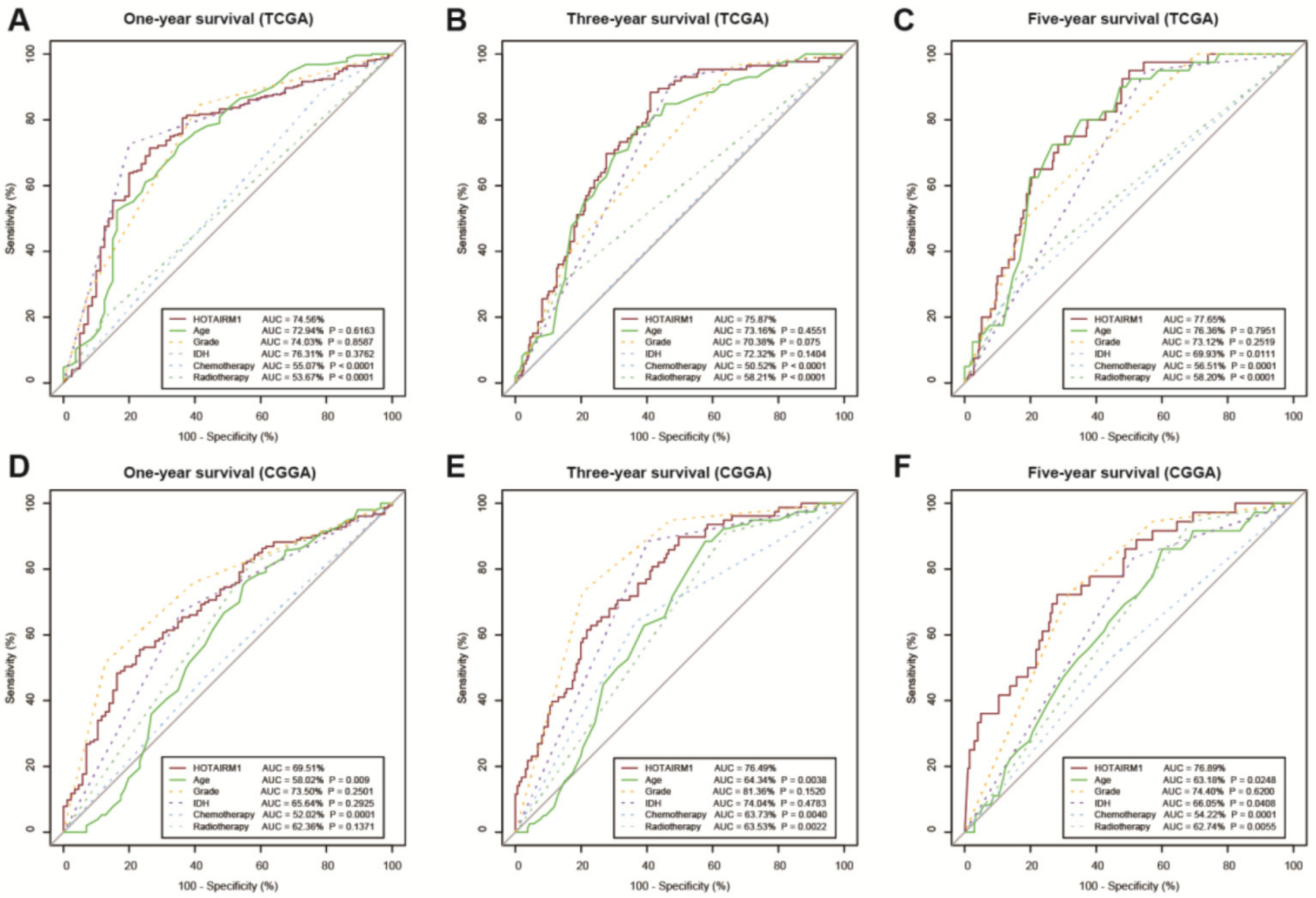

Supplementary Figure 3. HOTAIRM1 as a predictor of survival in glioma patients. HOTAIRM1 expression levels predicted one-year, three-year, and five-year survival in TCGA (**A**–**C**) and CGGA (**D**–**F**).

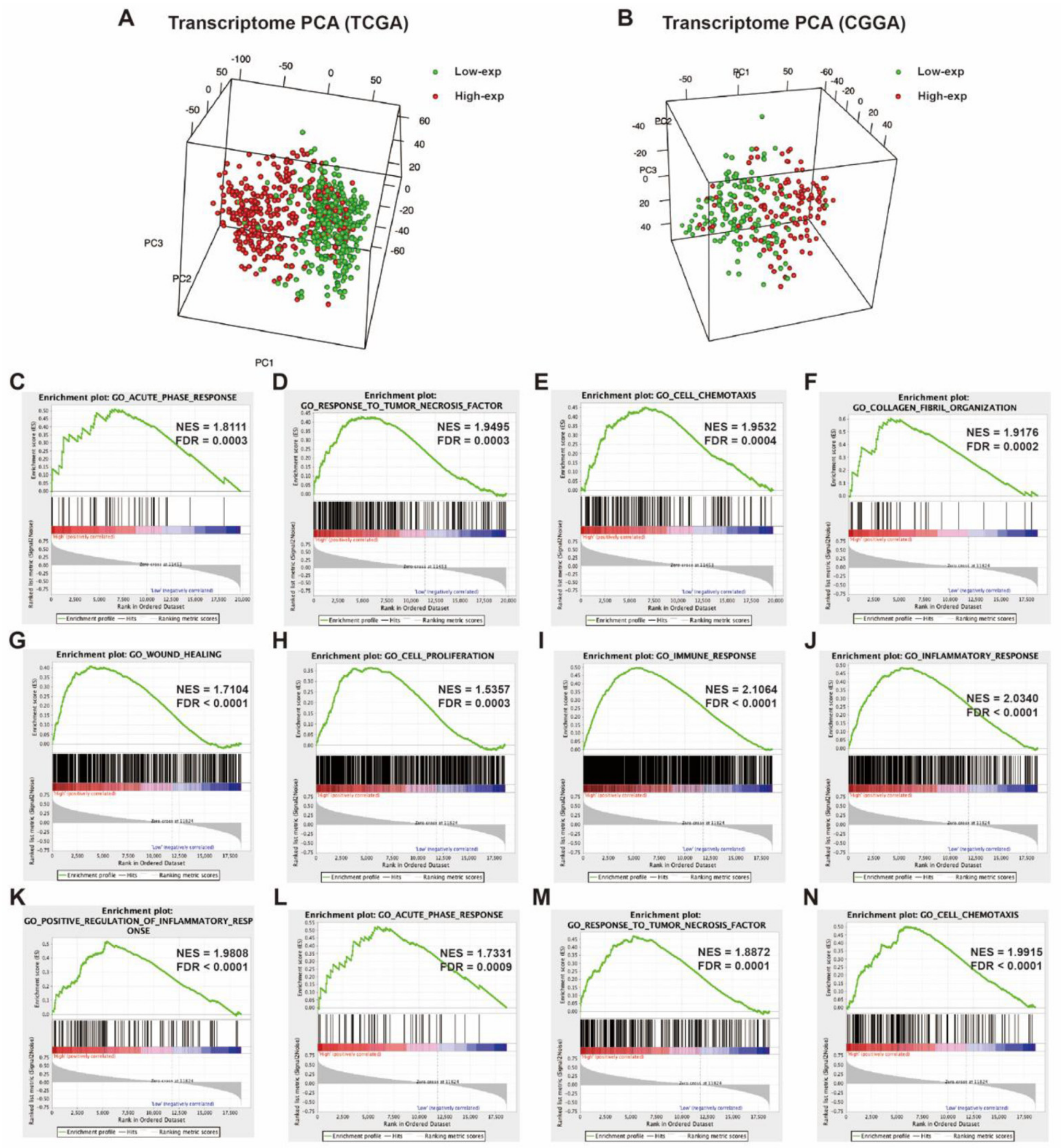

Supplementary Figure 4. Functional analysis of HOTAIRM1 in glioma. HOTAIRM1 was closely correlated with transcriptome expression profiles in TCGA (**A**) and CGGA (**B**). Biologic functions of HOTAIRM1 were confirmed by GSEA in TCGA (**C-E**) and CGGA (**F-N**). High-exp, high expression of HOTAIRM1; Low-exp, low expression of HOTAIRM1.

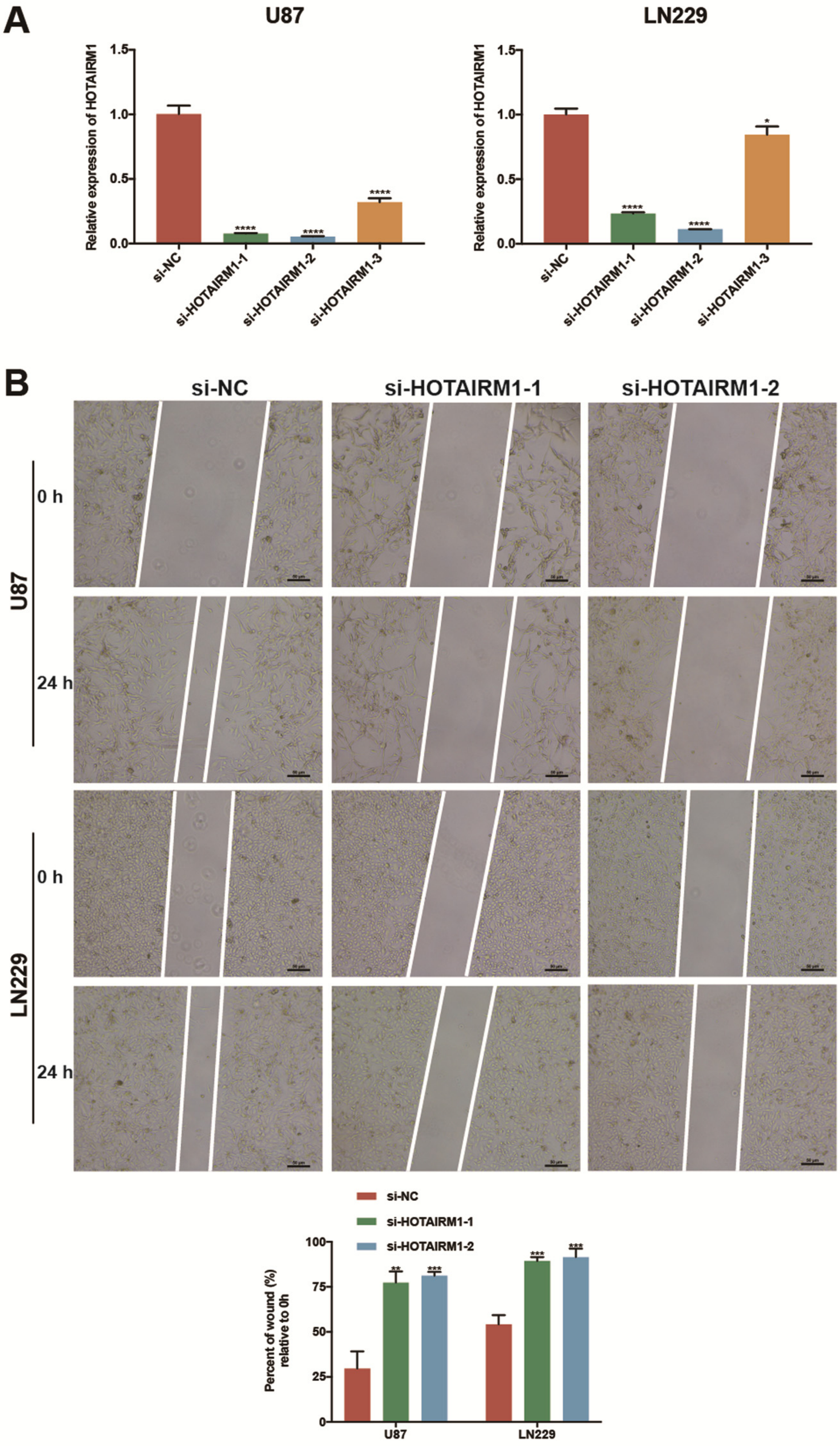

Supplementary Figure 5. HOTAIRM1 promotes cell migration *in vitro*. (**A**) Knockdown efficiency of si-HOTAIRM1-1, -2, and -3 in U87 and LN229 cells assessed by qRT-PCR. (**B**) Wound-healing assays were performed to evaluate the effect of si-HOTAIRM1 on cell migration. *p < 0.05, **p < 0.01, ***p < 0.001, ****p < 0.0001, values represented as the mean ± SD, Student's t-test.

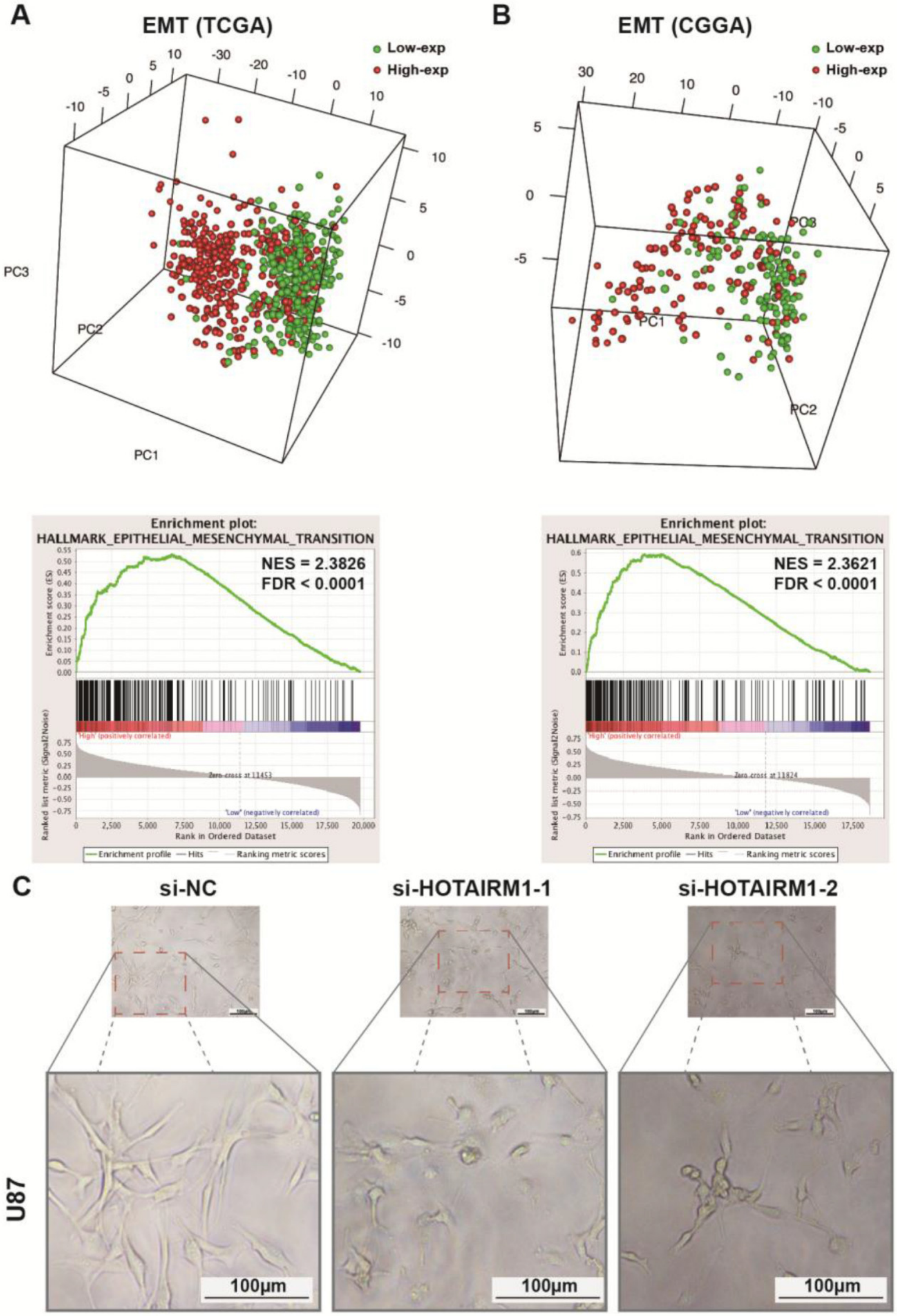

Supplementary Figure 6. HOTAIRM1 was correlated with EMT. (**A**) EMT differed between the high-exp and low-exp groups in TCGA and CGGA. (**B**) GSEA was performed to validate the effect of HOTAIRM1 on EMT in TCGA and CGGA. (**C**) Photomicrograph of U87 ten days after first transfection (×20 magnification; second transfection was performed five days after first transfection to maintain silence efficiency). EMT, epithelial-mesenchymal transition. High-exp, high expression of HOTAIRM1; Low-exp, low expression of HOTAIRM1.

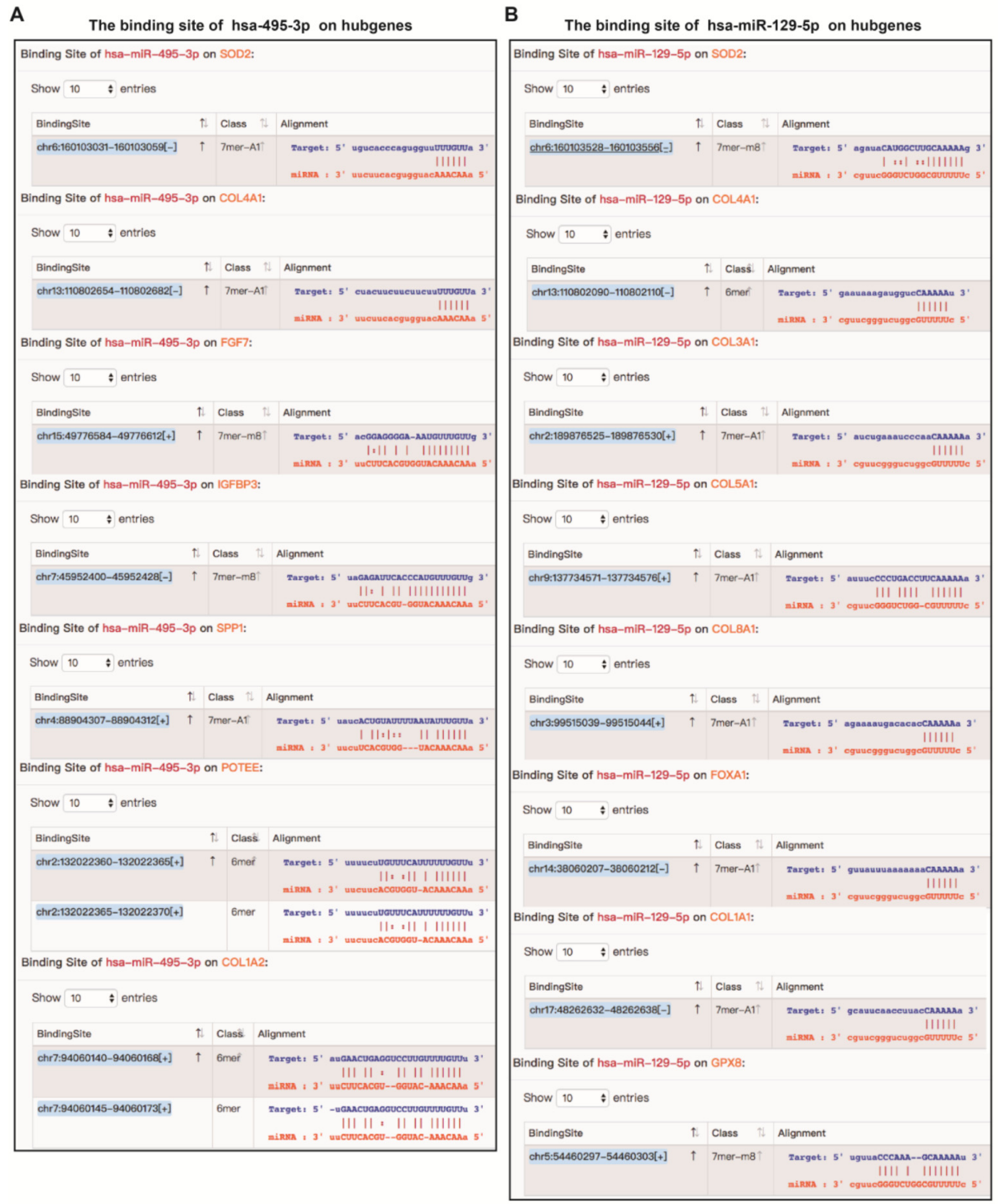

Supplementary Figure 7. The binding site of hsa-miR-495-3p and hsa-miR-129-5p on hub genes predicted with starBase v3.0 (http://starbase.sysu.edu.cn).

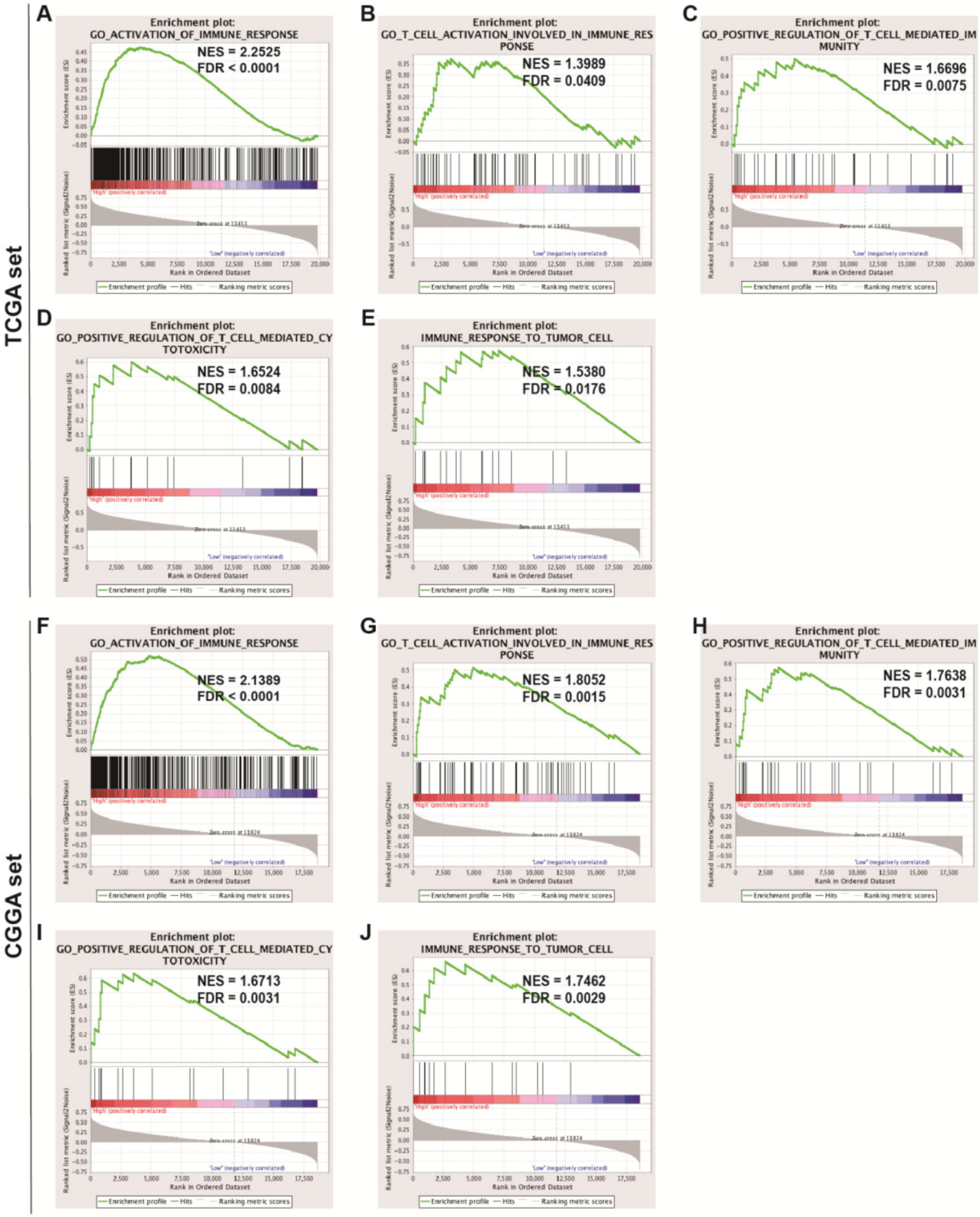

Supplementary Figure 8. Patients with high HOTAIRM1 expression showed a clear immune phenotype. GSEA was performed to confirm the effect of HOTAIRM1 on immune-related gene sets in TCGA (**A**–**E**) and CGGA (**F**–**J**).

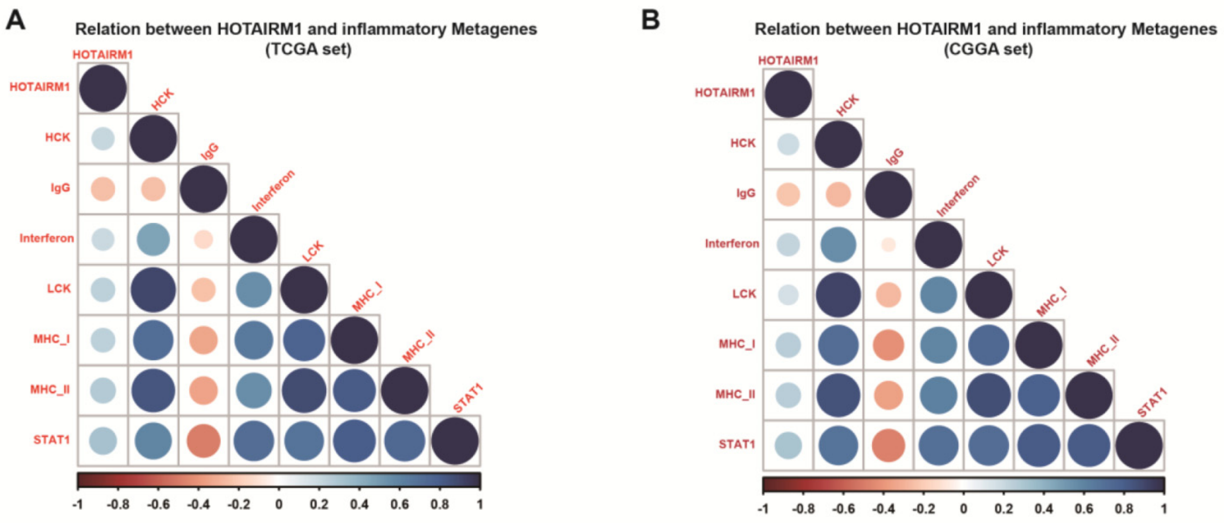

Supplementary Figure 9. HOTAIRM1 was associated with the regulation of inflammatory activities. Effect of HOTAIRM1 on inflammatory activities in TCGA (**A**) and CGGA (**B**) was assessed by Pearson correlation analysis.

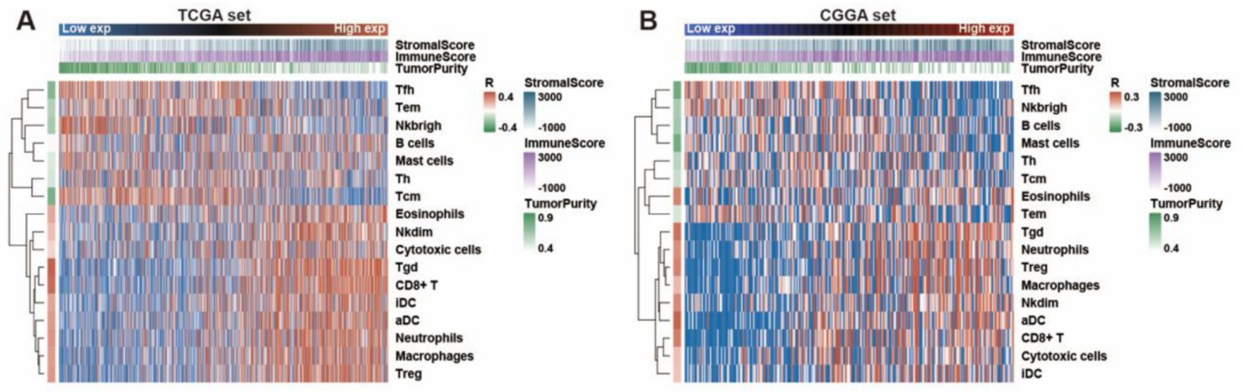

Supplementary Figure 10. HOTAIRM1 was associated with the regulation of the tumor microenvironment (TME). (**A**, **B**) High HOTAIRM1 expression was closely associated with low purity and immune cells including Gamma delta T cells (Tgd), CD8+ T cells, activated dendritic cells (aDC), and Regulatory T cells (Treg).

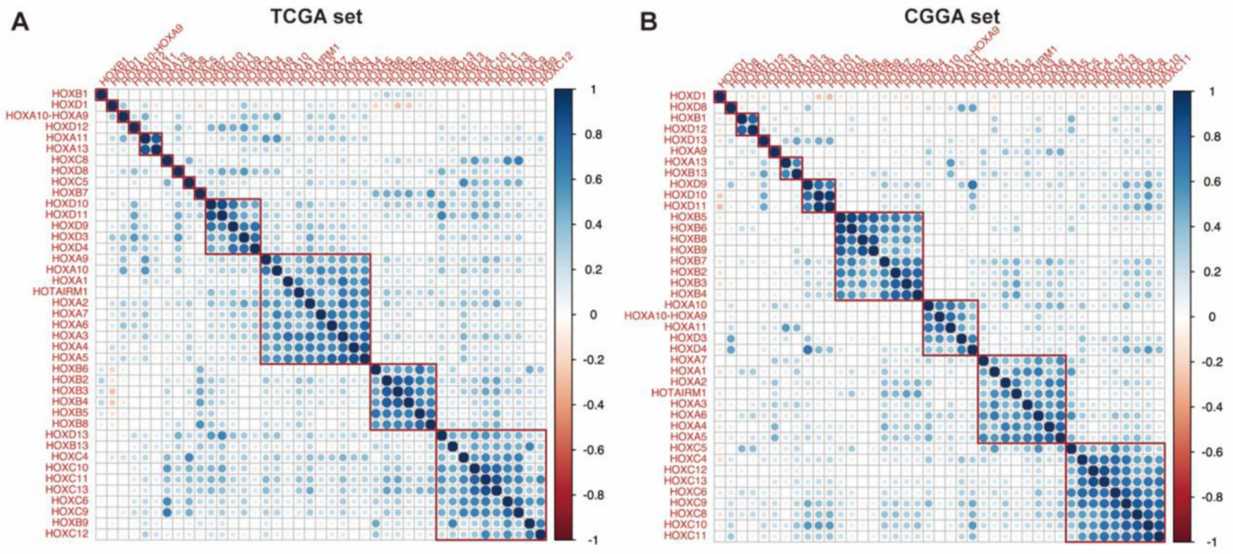

Supplementary Figure 11. HOTAIRM1 is positively associated with the HOXA gene family. (**A**, **B**) The relation between HOTAIRM1 and HOX gene family was investigated by Pearson's correlation analysis.
