# Supplementary material for: Long non-coding RNA, HOTAIRM1, promotes glioma malignancy by forming a ceRNA network
Source: Aging (Albany NY). 2019 Sep 2;11(17):6805–38. doi: 10.18632/aging.102205 (PMC6756894; doi:10.18632/aging.102205)
Supplement: Supplementary Tables [file aging-11-102205-s004.pdf]

SUPPLEMENTARY TABLES

Supplementary Table 1. Clinical and molecular information of glioma samples from TCGA and CGGA set included in this study.

	TCGA set (N = 672)	CGGA set (N = 274)
**Age, years**		
Mean (range)	46.86 (14-89)	42.92 (8-81)
**Gender**		
Female	284 (42.3%)	105 (38.3%)
Male	386 (57.4%)	169 (61.7%)
Unavailable	2 (0.3%)	0 (0.0%)
**KPS**		
Mean (range)	83.52 (40-100)	Unavailable
**WHO grade**		
Grade II	216 (32.1%)	101 (36.9%)
Grade III	237 (35.3%)	72 (26.3%)
Grade IV	160 (23.8%)	101 (36.9%)
Unavailable	59 (8.8%)	0 (0.0%)
**Histopathology**		
Oligodendroglioma	190 (28.3%)	34 (12.4%)
Oligoastrocytoma	128 (19.0%)	72 (26.3%)
Astrocytoma	192 (28.6%)	67 (24.5%)
Glioblastoma	160 (23.8%)	101 (36.9%)
Unavailable	2 (0.3%)	0 (0.0%)
**Molecular classification**		
LGG-Oligo	175 (26.0%)	0 (0.0%)
LGG-Astro	239 (35.6%)	0 (0.0%)
LGG-IDHwt	94 (14.0%)	48 (17.5%)
GBM-IDHmut	11 (1.6%)	27 (9.9%)
GBM-IDHwt	143 (21.3%)	74 (27.0%)
Unavailable	10 (1.5%)	125 (45.6%)
**TCGA subtype**		
Classical	214 (31.8%)	55 (20.1%)
Mesenchymal	59 (8.8%)	54 (19.7%)
Neural	28 (4.2%)	74 (27.0%)
Proneural	371 (55.2%)	91 (33.2%)
**IDH status**		
Mutant	425 (63.2%)	152 (55.5%)
Wild-type	238 (35.4%)	122 (44.5%)
Unavailable	9 (1.3%)	0 (0.0%)
**1p19q status**		
codel	168 (25.0%)	0 (0.0%)
non-codel	498 (74.1%)	0 (0.0%)
Unavailable	6 (0.9%)	274 (100.0%)
**Chr7 gain/Chr10 loss**		
YES	156 (23.2%)	0 (0.0%)
NO	507 (75.4%)	0 (0.0%)
Unavailable	9 (1.3%)	274 (100.0%)
**MGMT promoter status**		
Methylated	474 (70.5%)	0 (0.0%)
Unmethylated	164 (24.4%)	0 (0.0%)

Unavailable	34 (5.1%)	274 (100.0%)
**TERT promoter status**		
Mutant	157 (23.4%)	0 (0.0%)
Wild-type	163 (24.3%)	0 (0.0%)
Unavailable	352 (52.4%)	274 (100.0%)
PTEN status		
Mutant	64 (9.5%)	13 (4.7%)
Wild-type	588 (87.5%)	74 (27.0%)
Unavailable	20 (3.0%)	187 (68.2%)
**ATRX status**		
Mutant	195 (29.0%)	30 (10.9%)
Wild-type	463 (68.9%)	244 (89.1%)
Unavailable	14 (2.1%)	0 (0.0%)
**TP53 status**		
Mutant	280 (41.7%)	163 (59.5%)
Wild-type	372 (55.4%)	111 (40.5%)
Unavailable	20 (3.0%)	0 (0.0%)
**EGFR status**		
Mutant	66 (9.8%)	100 (36.5%)
Wild-type	586 (87.2%)	174 (63.5%)
Unavailable	20 (3.0%)	0 (0.0%)
**Radiotherapy**		
YES	413 (61.5%)	183 (66.8%)
NO	198 (29.5%)	72 (26.3%)
Unavailable	61 (9.1%)	19 (6.9%)
**Chemotherapy**		
YES	344 (51.2%)	135 (49.3%)
NO	52 (7.7%)	113 (41.2%)
Unavailable	276 (41.1%)	26 (9.5%)
**Overall survival, months**		
Median	29.3	43.2
**Survival status**		
Alive	248 (36.9%)	118 (43.1%)
Dead	417 (62.1%)	141 (51.5%)
Unavailable	7 (1.0%)	15 (5.5%)

Supplementary Table 2. The primers and microRNA assays in this study.

Primer name	Primer sequence (5'-3') or assay ID
HOTAIRM1 Fwd	CTGGCGAGAGGTCTGTTTTG
HOTAIRM1 Rev	AACACCCACATTTCAACCCC
FOXA1 Fwd	GCAATACTCGCCTTACGGCT
FOXA1 Rev	TACACACCTTGGTAGTACGCC
COL1A1 Fwd	GAGGGCCAAGACGAAGACATC
COL1A1 Rev	CAGATCACGTCATCGCACAAC
COL3A1 Fwd	TTGAAGGAGGATGTTCCCATCT
COL3A1 Rev	ACAGACACATATTTGGCATGGTT
COL8A1 Fwd	GGGAGTGCTGCTTACCATTTC
COL8A1 Rev	AGCGGCTTGATCCCATAGTAG
GPX8 Fwd	TACTTAGGGCTGAAGGAACTGC
GPX8 Rev	GGCTCCGATTCTCCAAACTGA
SOD2 Fwd	GCTCCGGTTTTGGGGTATCTG
SOD2 Rev	GCGTTGATGTGAGGTTCCAG
COL5A1 Fwd	TACAACGAGCAGGGTATCCAG
COL5A1 Rev	ACTTGCCATCTGACAGGTTGA
FGF7 Fwd	TCCTGCCAACTTTGCTCTACA
FGF7 Rev	CAGGGCTGGAACAGTTCACAT
IGFBP3 Fwd	AGACACACTGAATCACCTGAAGT
IGFBP3 Rev	AGGGCGACACTGCTTTTTCTT
COL1A2 Fwd	GAGCGGTAACAAGGGTGAGC
COL1A2 Rev	CTTCCCCATTAGGGCCTCTC
SPP1 Fwd	GCCGAGGTGATAGTGTGGTT
SPP1 Rev	AACGGGGATGGCCTTGTATG
COL4A1 Fwd	GGACTACCTGGAACAAAAGGG
COL4A1 Rev	GCCAAGTATCTCACCTGGATCA
POTEE Fwd	TGGATGATGATACCGCCGTG
POTEE Rev	TCCCAGTTGGTGATGATGCC
IL1B Fwd	ATGATGGCTTATTACAGTGGCAA
IL1B Rev	GTCGGAGATTCGTAGCTGGA
IL6 Fwd	ACTCACCTCTTCAGAACGAATTG
IL6 Rev	CCATCTTTGGAAGGTTCAGGTTG
TGFB1 Fwd	CTAATGGTGGAAACCCACAACG
TGFB1 Rev	TATCGCCAGGAATTGTTGCTG
PTGS2 Fwd	TAAGTGCGATTGTACCCGGAC
PTGS2 Rev	TTTGTAGCCATAGTCAGCATTGT
STAT3 Fwd	ACCAGCAGTATAGCCGCTTC
STAT3 Rev	GCCACAATCCGGGCAATCT
CCL2 Fwd	CAGCCAGATGCAATCAATGCC
CCL2 Rev	TGGAATCCTGAACCCACTTCT
18S Fwd	GCAGAATCCACGCCAGTACAAGAT
18S Rev	TCTTCTTCAGTCGCTCCAGGTCTT
hsa-miR-129-5p	Assay ID: 002298
hsa-miR-495-3p	Assay ID: 001663
U6	Assay ID: 001093

Supplementary Table 3. The distribution of clinical and molecular features between high-exp group and low-exp group.

	TCGA set			CGGA set		
	**High-exp group (N = 336)**	**Low-exp group (N = 336)**	**P value**	**High-exp group (N = 137)**	**Low-exp group (N = 137)**	**P value**
**Age, years**			<0.0001			<0.0001
Mean (range)	52.79 (21-89)	40.89 (14-75)		46.36 (8-81)	39.49 (10-	
**Gender**			0.0623			0.2140
Female	130 (38.7%)	154 (45.8%)		47 (34.3%)	58 (42.3%)	
Male	206 (61.3%)	180 (53.6%)		90 (65.7%)	79 (57.7%)	
Unavailable	0 (0.0%)	2 (0.6%)		0 (0.0%)	0 (0.0%)	
**KPS**			<0.0001			Unavailable
Mean (range)	79.61 (40-100)	88.31 (40-90)		Unavailable	Unavailable	
**WHO grade**			<0.0001			<0.0001
Grade II	45 (13.4%)	171 (50.9%)		25 (18.2%)	76 (55.5%)	
Grade III	110 (32.7%)	127 (37.8%)		34 (24.8%)	38 (27.7%)	
Grade IV	152 (45.2%)	8 (2.4%)		78 (56.9%)	23 (16.8%)	
Unavailable	29 (8.6%)	30 (8.9%)		0 (0.0%)	0 (0.0%)	
**Histopathology**			<0.0001			<0.0001
Oligodendroglio	44 (13.1%)	146 (43.5%)		6 (4.4%)	28 (20.4%)	
Oligoastrocytoma	44 (13.1%)	84 (25.0%)		24 (17.5%)	48 (35.0%)	
Astrocytoma	96 (28.6%)	96 (28.6%)		29 (21.2%)	38 (27.7%)	
Glioblastoma	152 (45.2%)	8 (2.4%)		78 (56.9%)	23 (16.8%)	
Unavailable	0 (0.0%)	2 (0.6%)		0 (0.0%)	0 (0.0%)	
**Molecular**			<0.0001			0.0003
LGG-Oligo	35 (10.4%)	140 (41.7%)		0 (0.0%)	0 (0.0%)	
LGG-Astro	73 (21.7%)	166 (49.4%)		0 (0.0%)	0 (0.0%)	
LGG-IDHwt	75 (22.3%)	19 (5.7%)		29 (21.2%)	19 (13.9%)	
GBM-IDHmut	7 (2.1%)	4 (1.2%)		14 (10.2%)	13 (9.5%)	
GBM-IDHwt	140 (41.7%)	3 (0.9%)		64 (46.7%)	10 (7.3%)	
Unavailable	6 (1.8%)	4 (1.2%)		30 (21.9%)	95 (69.3%)	
**TCGA subtype**			<0.0001			<0.0001
Classical	153 (45.5%)	61 (18.2%)		41 (29.9%)	14 (10.2%)	
Mesenchymal	57 (17.0%)	2 (0.6%)		49 (35.8%)	5 (3.6%)	
Neural	26 (7.7%)	2 (0.6%)		18 (13.1%)	56 (40.9%)	
Proneural	100 (29.8%)	271 (80.7%)		29 (21.2%)	62 (45.3%)	
**IDH status**			<0.0001			<0.0001
Mutant	115 (34.2%)	310 (92.3%)		44 (32.1%)	108 (78.8%)	
Wild-type	215 (64.0%)	23 (6.8%)		93 (67.9%)	29 (21.2%)	
Unavailable	6 (1.8%)	3 (0.9%)		0(0.0%)	0(0.0%)	
**1p19q status**			<0.0001			Unavailable
codel	33 (9.8%)	135 (40.2%)		0 (0.0%)	0 (0.0%)	
non-codel	298 (88.7%)	200 (59.5%)		0 (0.0%)	0 (0.0%)	
Unavailable	5 (1.5%)	1 (0.3%)		137 (100.0%)	137	
**Chr7 gain/Chr10 loss**			<0.0001			Unavailable
YES	150 (44.6%)	6 (1.8%)		0 (0.0%)	0 (0.0%)	
NO	181 (53.9%)	326 (97.0%)		0 (0.0%)	0 (0.0%)	
Unavailable	5 (1.5%)	4 (1.2%)		137 (100.0%)	137	
**MGMT promoter**			<0.0001			Unavailable
Methylated	179 (53.3%)	295 (87.8%)		0 (0.0%)	0 (0.0%)	
Unmethylated	125 (37.2%)	39 (11.6%)		0 (0.0%)	0 (0.0%)	

Unavailable	32 (9.5%)	2 (0.6%)		137 (100.0%)	137	
**TERT promoter**			0.0025			Unavailable
Mutant	75 (22.3%)	82 (24.4%)		0 (0.0%)	0 (0.0%)	
Wild-type	50 (14.9%)	113 (33.6%)		0 (0.0%)	0 (0.0%)	
Unavailable	211 (62.8%)	141 (42.0%)		137 (100.0%)	137	
**PTEN status**			<0.0001			0.0352
Mutant	60 (17.9%)	4 (1.2%)		11 (8.0%)	2 (1.5%)	
Wild-type	265 (78.9%)	323 (96.1%)		35 (25.5%)	39 (28.5%)	
Unavailable	11 (3.3%)	9 (2.7%)		91 (66.4%)	96 (70.1%)	
**ATRX status**			<0.0001			0.8466
Mutant	68 (20.2%)	127 (37.8%)		16 (11.7%)	14 (10.2%)	
Wild-type	257 (76.5%)	206 (61.3%)		121 (88.3%)	123 (89.8%)	
Unavailable	11 (3.3%)	3 (0.9%)		0 (0.0%)	0 (0.0%)	
**TP53 status**			0.0260			0.0850
Mutant	125 (37.2%)	155 (46.1%)		89 (65.0%)	74 (54.0%)	
Wild-type	200 (59.5%)	172 (51.2%)		48 (35.0%)	63 (46.0%)	
Unavailable	11 (3.3%)	9 (2.7%)		0 (0.0%)	0 (0.0%)	
**EGFR status**			<0.0001			0.2587
Mutant	61 (18.2%)	5 (1.5%)		45 (32.8%)	55 (40.1%)	
Wild-type	264 (78.6%)	322 (95.8%)		92 (67.2%)	82 (59.9%)	
Unavailable	11 (3.3%)	9 (2.7%)		0 (0.0%)	0 (0.0%)	
**Radiotherapy**			<0.0001			0.8867
YES	242 (72.0%)	171 (50.9%)		90 (65.7%)	93 (67.9%)	
NO	66 (19.6%)	132 (39.3%)		34 (24.8%)	38 (27.7%)	
Unavailable	28 (8.3%)	33 (9.8%)		13 (9.5%)	6 (4.4%)	
**Chemotherapy**			0.0080			0.0054
YES	187 (55.7%)	157 (46.7%)		75 (54.7%)	60 (43.8%)	
NO	39 (11.6%)	13 (3.9%)		48 (35.0%)	65 (47.4%)	
Unavailable	110 (32.7%)	166 (49.4%)		14 (10.2%)	12 (8.8%)	

For catergory features we used chi-square or Fisher's exact test. For continuous features we calculate p value by Student's t test.

Supplementary Table 4. The distribution features of HOTAIRM1 expression level in various glioma subtypes.

	TCGA set		CGGA set	
	Expression level of HOTAIRM1 (mean (sd))	P value	Expression level of HOTAIRM1 (mean (sd))	Pvalue
WHO grade		<0.0001		<0.0001
Grade II	0.45 (0.54)		0.50 (0.55)	
Grade III	1.00 (1.13)		1.14 (1.25)	
Grade IV	2.44 (1.15)		1.77 (1.15)	
Histopathology		<0.0001		<0.0001
Oligodendroglioma	0.52 (0.73)		0.37 (0.41)	
Oligoastrocytoma	0.65 (0.77)		0.78 (1.05)	
Astrocytoma	1.07 (1.18)		0.95 (1.00)	
Glioblastoma	2.44 (1.15)		1.77 (1.15)	
Molecular classification		<0.0001		0.0009
LGG-Oligo	0.40 (0.49)		Unavailable	
LGG-Astro	0.52 (0.47)		Unavailable	
LGG-IDHwt	2.03 (1.41)		1.38 (1.37)	
GBM-IDHmut	1.61 (1.03)		1.12 (0.96)	
GBM-IDHwt	2.52 (1.13)		2.01 (1.12)	
TCGA subtype		<0.0001		<0.0001
Proneural	0.63 (0.88)		0.70 (0.79)	
Classical	1.61 (1.33)		2.06 (1.47)	
Neural	2.44 (1.24)		0.55 (0.60)	
Mesenchymal	2.26 (0.99)		1.75 (0.92)	

P value was calculated by one-way ANOVA.

Supplementary Table 5. IC50 of TMZ in si-NC or si-HOTAIRM1 group.

	IC50 (μM) (U87)	IC50 (μM) (LN229)
**NC**	494.1	1367
**si-HOTAIRM1-1**	220.8	629.5
**si-HOTAIRM1-2**	200.7	751.3

Please browse Full Text version to see the data of Supplementary Datasets 1–3

Supplementary Dataset 1. Differentially upregulated genes in high-exp group compared with low-exp group.

Supplementary Dataset 2. Differentially downregulated miRNAs (down-DE miRNAs) in glioblastoma (GBM) compared with normal brain tissue.

Supplementary Dataset 3. Gene set on activation of immune response, T cell-mediated immune response and immune response to tumor cell.
